# Experimental Validation of Modeling Approaches Describing the Crystallization of Pure Triacylglycerides and Their Mixtures: A Review

**DOI:** 10.1111/1541-4337.70315

**Published:** 2025-10-24

**Authors:** Michele Lessona, Antoine Cros, Laurent Sagalowicz, Yogesh Harshe, Antonio Buffo, Elena Simone

**Affiliations:** ^1^ Department of Applied Science and Technology (DISAT) Politecnico di Torino Torino Italy; ^2^ Nestlé Research, Vers‐chez‐les‐Blanc Lausanne Switzerland; ^3^ School of Food Science and Nutrition, Food Colloids and Bioprocessing Group University of Leeds Leeds UK

## Abstract

Lipids are essential macronutrients, with the ability to solubilize and enhance the uptake of specific micronutrients (e.g., vitamins A, D, E, and K). Solid fats provide desirable texture and palatability to food products, making them a key ingredient for food and particularly bakery products, ice cream, or confectionery. Outside of the food industry, lipids are often used in pharmaceutical formulations to facilitate the delivery of poorly water‐soluble active pharmaceutical ingredients. Natural fats, such as cocoa butter or tallow, consist of a complex mixture of several triglycerides (TAGs) with different chemical compositions. Such differences can lead to partial or full immiscibility in the solid phase, which results in complex phase diagrams with multiple possible types of solid phases. Additionally, TAGs can crystallize in many crystalline forms (polymorphs) with different thermodynamic stabilities, which further complicates TAG solid‐state thermodynamic and kinetic behavior. Due to this complexity, developing experimentally validated models that describe the crystallization behavior of TAG mixtures is challenging. Nevertheless, reliable models are essential to speed up product and process development and increase manufacturing efficiency. This review paper illustrates and discusses existing models developed to describe different aspects of TAGs crystallization, with a focus on their experimental validation and the necessary analytical tools. For all approaches and experimental techniques, advantages and limitations are presented. Finally, based on the reviewed literature, possible future research trends and developments are presented.

## 1 Introduction to Fats and Oils and Their Crystallization Behavior

Fats are a fundamental macronutrient commonly used for home cooking, in the food industry for their flavor, texture, functionalities, and for their food‐preserving properties. Compared with carbohydrates and proteins, they have a higher energy density, contain essential micronutrients such as omega‐3 and omega‐6 fatty acids, and solubilize lipophilic vitamins (Lewis 2014). Depending on their physical state at room temperature, it is possible to distinguish between fats (solid) and oils (liquid). Seeds, legumes, nuts, dairy, and meat are common fat‐rich food products (Foster et al. 2009).

Edible fats are mixtures of several triacylglycerols (TAGs). From a chemical point of view, a TAG molecule is a triester made of a glycerol backbone and three fatty acid (FAs) chains. Monoacid TAGs contain only one type of fatty acid, whereas diacid or triacid TAGs contain two or three different FAs. Diacid TAGs can be symmetrical or asymmetrical depending on the arrangement of the FA chains in the glycerol backbone (Bayés‐García et al. 2020; Marangoni and Wesdorp 2012).

FA can be fully saturated, mono‐ or polyunsaturated; unsaturated TAGs can show cis or trans geometric isomerism of the carbon‐carbon double bond; this spatial orientation deeply affects molecular packing and crystallization. Natural fats usually present a complex and diverse TAG profile, depending on the source of origin. In fact, significant differences in TAGs composition can be observed in animal fats originating from different species (e.g., cow vs. buffalo milk fat), or in plant‐based fats harvested at different times of the year or of different geographical origins (Marty‐Terrade and Marangoni 2012; van Malssen et al. 1996).

Demand for oils and fats has been steadily increasing worldwide. Edible oil production increased by 48% from 1995 to 2011 (Parcell et al. 2018), and it is anticipated to grow yearly by 3.3% from 2020 to 2027 (Breaking Down Fats and Oils 2021) in developing countries. This poses new challenges for producers, who also need to account for environmental, geopolitical, socioeconomic, and social factors that can directly influence the demand, the availability, and the price of natural fats and oils (Breaking Down Fats and Oils 2021; Parcell et al. 2018). For these reasons, finding new sources of oils and fats and understanding how they can be used to fully or partly replace the currently used ones is essential.

Fats are widely used as ingredients in the food industry due to their sensory, textural, and structuring properties; these are strongly related to the properties of fat crystals (size, shape, polymorphism). Hence, understanding the crystallization behavior of complex TAGs is essential for the development of novel fat‐based food products.

TAGs in the crystalline state arrange themselves in two types of conformations called chair or tuning fork, based on the relative position of the different fatty chains in relation to the glycerol backbone. (Acevedo and Marangoni 2015; Jensen and Mabis 1966) In the chair configuration, fatty chains in positions sn‐2 and sn‐3 are parallel to each other, while in the tuning‐fork conformation, the parallel chains are those in positions sn‐1 and sn‐3.

**FIGURE 1:**
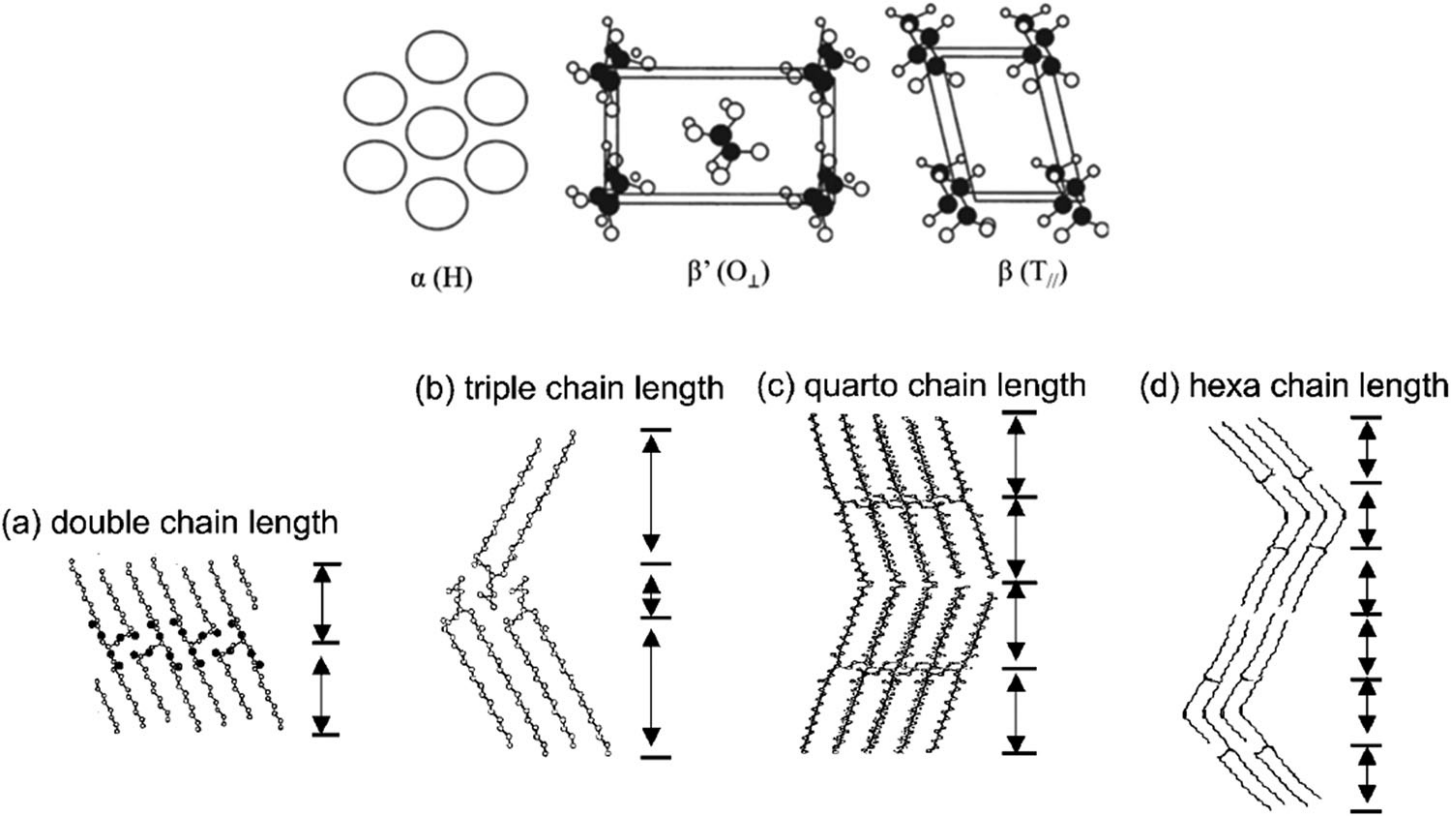
Top: crystal subcell of FA lateral packing for the three different main TAG polymorphs. Bottom: (a) long spacing in a 2L lamellar stacking, (b) short spacing in a β subcell. *Source*: Adapted from Bayés‐García et al. (2020).

Crystallizing TAGs stack side by side, forming lamellar phases. Stacking can happen in several ways, and structures with double (2L), triple (3L), quatro (4L), or hexa (6L) chain lengths can form. L refers to the generic fatty acid length, and the number indicates the number of chains in between the two ending methyl groups in the lamella (Figure 1). The distance between these groups is commonly referred to as long d‐spacing. (Bayés‐García et al. 2020; Goto et al. 1992; Jensen and Mabis 1966; Van Langevelde et al. 2000). TAGs can crystallize in several polymorphs, each of which presents different physical properties (e.g., melting point, mechanical strength). The most common fat polymorphs are alpha (α), beta prime (β′), and beta (β), in order of increasing thermodynamic stability, density, and melting point, respectively. The main structural difference between polymorphs is the lateral fatty acid packing in the crystal subcell. The α polymorph presents a hexagonal (H) subcell, β′ presents an orthorhombic O_⊥_ subcell, and the most stable and dense β form presents a triclinic T*_||_* subcell, as shown in Figure 1 (Idziak 2018; Larsson et al. 1966; Sato 2001). The Gibbs free energy of the α, β′, and β polymorphs of the same TAG is different, with the β structure having the lowest value (i.e., most thermodynamically stable form); additionally, these three polymorphic forms are monotropically related, meaning that a polymorphic transformation can only happen from a less stable to a more stable polymorph at any range of pressure and temperature.

This transformation can happen in the solid state or involve a melting and a recrystallization process (melt‐mediated). The crystallization driving force is the degree of supercooling, which is the difference between the lowest temperature reached by the liquid phase of the fat mixture and the melting point. A supercooled TAG mixture before nucleation is in a metastable condition; eventually, nuclei will begin to form, and crystallization will start. Normally, more stable polymorphs have higher activation energy for nucleation. This is the reason why the less thermodynamically stable α form is generally the first one to form, especially in high undercooling conditions. Over time, a conversion to the more stable β′ and then β polymorphs will occur (Hernqvist and Larsson 1982; Sato and Kuroda 1987).

TAG polymorphism directly influences important physical properties of fat‐based products, such as density, mechanical strength, and melting point, which in turn have an effect on product functionality, particularly texture and mouthfeel. Polymorphism also impacts the stability and the shelf‐life of these products; the fat bloom phenomenon is an example of an undesirable polymorphic transformation of the main fat ingredient in chocolate, cocoa butter (Ghazani and Marangoni 2021). Since only specific polymorphs are desirable in fat‐based food products such as chocolate, ice cream, or margarine, a good knowledge of the polymorphic landscape, the kinetic and thermodynamic behavior of TAG mixtures is essential. For a rational design of fat‐based food products, understanding the relationship between these parameters and the TAGs composition is particularly critical.

Natural fat crystallization is a complex process due to both the diverse TAG composition and the intrinsic polymorphism of fat mixtures; building reliable theoretical models and validating them with data from carefully designed experiments is of great value for the effective design of novel foods and for the reformulation of more sustainable existing fat‐based products.

Models reported in the literature are mainly based on three different approaches: thermodynamic, kinetic, and molecular; this distinction is also applied in this review, where each section is dedicated to the description of a modeling approach. Table 1 briefly summarizes the models that will be presented.

**TABLE 1:** Literature models analyzed in this work.

Author (year)	Type	Description
Timms (1984)	Thermodynamic	Mixed TAG model—linear combination of TAG components
Ollivon and Perron (1982)	Thermodynamic	Pure TAG model—linear combination of FAs contribution
Wesdorp (1990)**/**Marangoni and Wesdorp (2012)	Thermodynamic	Pure TAG model—FAs length, unsaturation, position
Zéberg‐Mikkelsen and Stenby (1999)	Thermodynamic	Pure TAG model—FAs symmetry
Seilert and Flöter (2021)	Thermodynamic	Pure TAG model—structure‐based model
Wesdorp (1990)/Marangoni and Wesdorp (2012)	Thermodynamic	Mixed TAG model—Margules model for nonideality
Brinkmann et al. (2019)	Thermodynamic	Mixed TAG model—PC‐SAFT
Pereira et al. (2019)	Thermodynamic	Mixed TAG model—UNIFAC
Marangoni et al. (2023)	Thermodynamic	Mixed TAG model—solid medium interaction
Schaink (2023)	Thermodynamic	Mixed TAG model—Hildebrand equation
Avrami (1940)	Kinetic	Avrami model
Khanna and Taylor (1988)	Kinetic	Modified Avrami model
Gompertz (1825)	Kinetic	Gompertz model
Foubert et al. (2002)	Kinetic	Kinetics as *n* ^th^ order forward reaction—1^st^ order backward reaction
Burton et al. (1951)	Kinetic	Burton Cabrera Frank model
Miyagawa et al. (2018)	Kinetic	Autocatalytic kinetic
Brasiello et al. (2011)	Molecular	Tridecanoin CG mapping
Pizzirusso et al. (2015)	Molecular	Tripalmitin and tristearin mixture crystallization
Cordina et al. (2023a)	Molecular	Mapping unsaturated TAGs
Cordina et al. (2023c)	Molecular	Melting of pure TAG
Cordina et al. (2023b)	Molecular	Melting of binary TAG mixtures

Each modeling approach is characterized by different objectives. Thermodynamic modeling focuses on the equilibrium thermal behavior of fat systems, which practically translates into the prediction of: (1) the phase diagrams of complex fat mixtures, from which it is possible to obtain (2) the solid–liquid equilibrium (SLE) lines and (3) the solid fat content (SFC). The kinetic modeling can describe (1) fat crystal growth and (2) the polymorphic behavior during the crystallization process and its evolution over time, which can be directly applied to the design of (3) the shelf life of a fat‐based product. Molecular modeling is a powerful tool that can shed light on the crystallization process from a molecular point of view by simulating the actual triglyceride packing in a fat crystal system.

Nevertheless, every model developed so far presents several limitations; this is due to the multiscale nature of the phenomenon of fat crystallization, and our poor knowledge of some key mechanisms, such as primary and secondary nucleation, and polymorphic transformation. A combination of different modeling techniques and experiments carried out with multiple analytical techniques is hence necessary for a better understanding of fat crystallization. An exception to this was reported by Hjorth et al. (2015a, 2015b), who investigated a TAG mixture crystallization process from both a thermodynamic and kinetic point of view.

On a final note, this review aims to provide a critical overview of the modeling approaches and the validation experiments used to describe fat crystallization at the sub‐micron scale. Hence, this review will not tackle secondary crystallization phenomena such as crystal agglomeration and ripening that are responsible for the development of the fat mixture microstructure (or fat crystal network) and its rheological properties. For these topics, the readers are invited to consult more specific literature such as (Macias‐Rodriguez and Marangoni 2018; Marangoni and Rousseau 1996; Narine and Marangoni 1999).

## 2 Thermodynamic Models of Fat Mixtures

### 2.1 Introduction to Thermodynamic Modeling

This section will focus on the thermodynamic modeling of TAG mixtures, which enables the prediction of the phase diagram of mixtures of two or more TAGs. From this information, it is then possible to estimate the Solid Fat Content (SFC) of such mixtures, which describes how the percentage of solid material varies with temperature (Himawan et al. 2006). While this characterization is useful for describing the thermal behavior of a fat mixture, it does not give any information on the size and morphology of the fat crystal network.

Solid–liquid equilibrium phase diagrams provide information on the number of phases existing at different temperatures and their TAG compositions. Such information is essential for the effective design of fractionation processes, which are widely applied to palm oil and milk fat to obtain multiple TAG mixtures with different thermal properties for multiple uses. However, obtaining phase diagrams of complex mixtures of TAGs is experimentally difficult. In fact, commonly used TAG mixtures such as milk fat are characterized by hundreds of different TAGs (Pratama et al. 2021), whereas the relatively simple cocoa butter presents three main TAGs, POP, POS, and SOS (Ghazani and Marangoni 2021) that constitute up to 80% of the total TAGs, and several other TAGs that can affect crystallization behavior (Simone et al. 2024).

**FIGURE 2:**
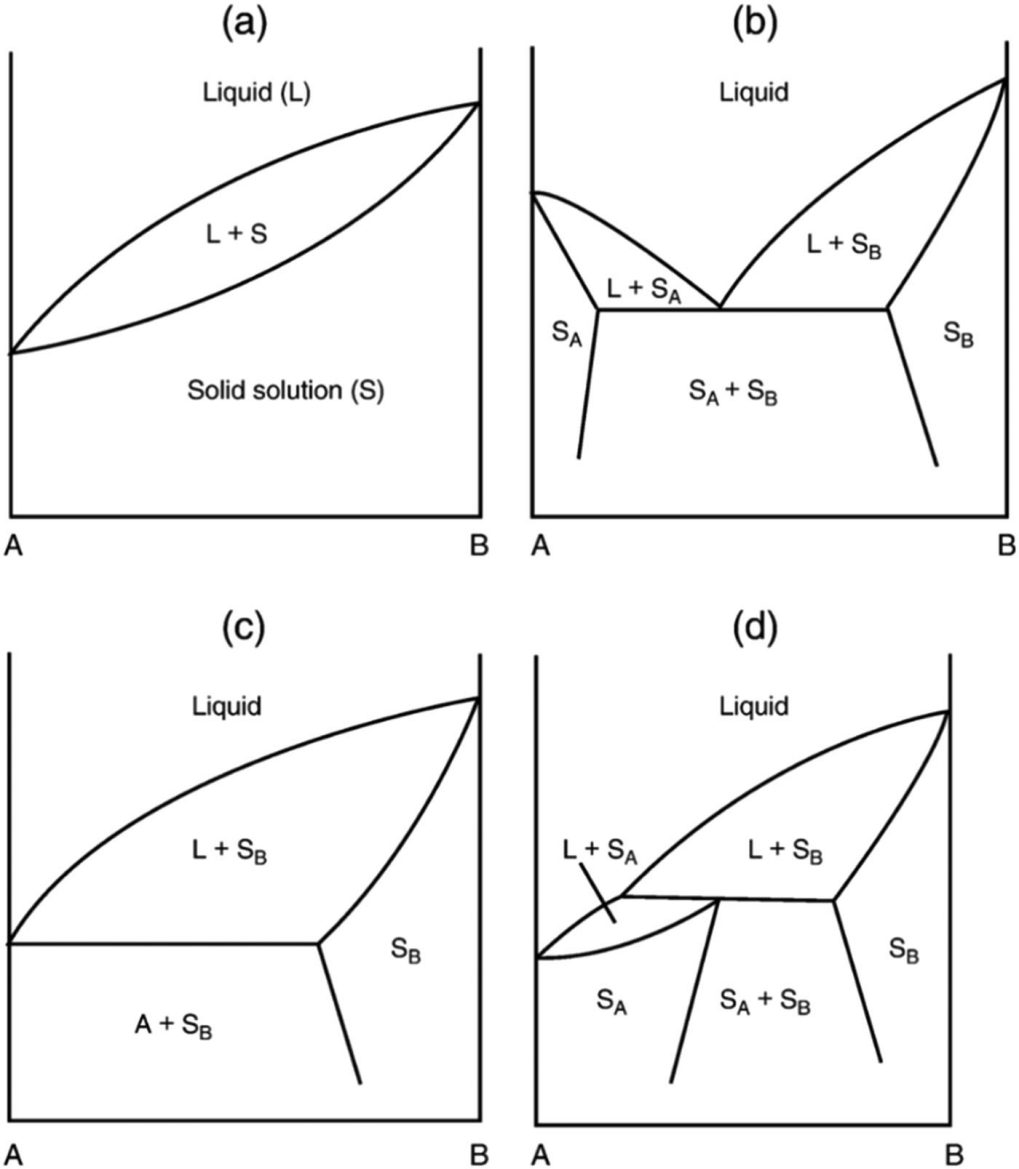
Most common binary phase behavior of TAGs. (a) Monotectic continuous solid solution, (b) eutectic, (c) monotectic partial solid solution, (d) peritectic. *Source*: Adapted from Timms (1984).

Solid phases in TAG mixtures may exhibit different phase behaviors: solid solutions, eutectic mixtures, and molecular compounds. Moreover, the presence of polymorphs with different thermodynamic stability adds to the total complexity of this representation (Bayés‐García et al. 2020; Costa et al. 2009; Maximo et al. 2014; Sasaki et al. 2012). Figure 2 (Timms 1984) shows the possible phase behaviors of binary TAG mixtures.

Several attempts have been made to develop models for phase diagrams in the last 40 years, but a comprehensive thermodynamic model that describes TAGs solid—liquid equilibria is still far from being achieved despite its importance in understanding the melting behavior of natural fat mixtures. The following paragraph outlines the theoretical basis and the progress of models previously used to describe the thermodynamic behavior of fat mixtures.

### 2.2 Types and Theoretical Principles of Existing Thermodynamic Models

Estimating the phase diagram of a TAGs mixture is not trivial: melting temperature and enthalpy of all polymorphs for each TAG must be known; this information needs to be measured experimentally or predicted by theoretical models. Knowing the exact composition of a mixture is fundamental information for every modeling approach. Information on the TAGs composition of a mixture can be obtained by analytical techniques such as chromatography, as detailed more specifically later in this review. However, together with the TAGs composition, it is also necessary to account for the nonideal interactions between TAG species in the mixture to correctly describe their mixing behavior. Several models have been developed over the years to obtain thermodynamic data for pure TAGs, as well as the estimation of nonideal interactions in a binary TAGs mixture. These models will be described in this section.

An early attempt to predict the SFC of fat mixtures was reported by Timms (1984), who criticized previous linear approaches and proposed two nonlinear methods to estimate melting temperature and SFC of fat blends. Even though those two methods refer to fat blends and not strictly TAG mixtures, those methods calculate ternary mixtures’ properties as a sum of a linear and an excess contribution, the latter calculated by pairwise interactions fitted on binary mixtures measurements. This method worked fairly well in estimating blends’ melting points but showed unreliable predictions for SFC. Compared with linear regression, these methods can be extended to new components only by measuring the new binary blends’ interactions. Despite this flexibility, fat blends models do not capture TAG level crystallization or polymorphism (Marangoni and Wesdorp 2012; Timms 1984).

Ollivon & Perron (Ollivon and Perron 1982) proposed a method to predict enthalpies of pure TAGs using a linear fit based on the total (all three FA) carbon atoms of the TAG molecule. Different equations were obtained for the three main TAGs polymorphs. This approach has the drawback of not considering the influence of the FA position on the glycerol backbone, which is known to influence the crystallization. Moreover, unsaturated TAGs and less common polymorphs are not taken into account.

Wesdorp (Marangoni and Wesdorp 2012; Wesdorp 1990a) developed one of the first and most comprehensive thermodynamic models describing fat crystallization. In principle, it could describe any kind of n components TAG mixture if the melting enthalpy and melting temperature of the pure TAGs, together with the pairwise activity coefficients, are known. This model is based on the thermodynamic definition of phase equilibrium (Equation 1):

(1) $$\mu_{i}^{\mathrm{solid}}=\mu_{i}^{\mathrm{liquid}},$$

where $\mu_{i}^{\mathrm{solid}}$ and $\mu_{i}^{\mathrm{liquid}}$ are the chemical potentials of the solid and liquid phases of the $i^{\mathrm{th}}$ TAG component in the mixture. They can be expressed as the sum of a pure ($\mu_{i}^{0})\;$ and an excess contribution that depends on the temperature T, the universal gas constant R, the fraction of the $i^{th}$ component in the solid and in the liquid ($x_{i}^{S}$ and $x_{i}^{L}$, respectively) and their respective activity coefficients $\gamma_{i}^{S}$ and $\gamma_{i}^{L}$:

(2) $$\mu_{i}^{0,S}+RT\ln\left(\gamma_{i}^{S}x_{i}^{S}\right)=\mu_{i}^{0,L}+RT\ln\left(\gamma_{i}^{L}x_{i}^{L}\right).$$

Equation (2) can be rewritten as Equation (3):

(3) $$RT\ln\left(\frac{\gamma_{i}^{S}x_{i}^{S}}{\gamma_{i}^{L}x_{i}^{L}}\right)=\left(\mu_{i}^{0,L}-\mu_{i}^{0,S}\right).$$

The difference between chemical potentials can also be expressed by $G_{L,i}-G_{S,i}$, which is the Gibbs free energy of the liquid L and the solid S for pure component i. Since TAGs are normally investigated at constant pressure, we can express the Gibbs free energy as G=H−TS where H is the enthalpy and S is the entropy, so Equation (3) becomes Equation (4):

(4) $$RT\ln\left(\frac{\gamma_{i}^{S}x_{i}^{S}}{\gamma_{i}^{L}x_{i}^{L}}\right)=H_{L,i}-H_{S,i}-T\left[S_{L,i}-S_{S,i}\right].$$

Differences in entropy and enthalpy can be further expanded as expressed by Equation (5):

(5) $$\begin{array}{ccc} RT\ln\left(\frac{\gamma_{i}^{S}x_{i}^{S}}{\gamma_{i}^{L}x_{i}^{L}}\right) & = & \Delta H_{m,i}+\Delta c_{p,i}\left(T_{i}-T_{m,i}\right)\\ & & -\;T\left[\frac{\Delta H_{m,i}}{T_{m,i}}+\Delta c_{p,i}\ln\left(\frac{T}{T_{m,i}}\right)\right]. \end{array}$$

Then, Equation (6) can be obtained by dividing by RT and rearranging:

(6) $$\begin{array}{ccc} \ln\left(\frac{\gamma_{i}^{S}x_{i}^{S}}{\gamma_{i}^{L}x_{i}^{L}}\right) & = & \frac{\Delta H_{m,i}}{R}\left(\frac{1}{T}-\frac{1}{T_{m,i}}\right)-\frac{\Delta c_{p,i}}{R}\left(\frac{T_{m,i}-T}{T}\right)\\ & & +\;\frac{\Delta c_{p,i}}{R}\ln\left(\frac{T_{m,i}}{T}\right), \end{array}$$

$\Delta H_{m,i}$ and $T_{m,i}$ are the melting enthalpy and the melting temperature, respectively, of the $i^{th}$ component and $\Delta c_{p,i}$ is the difference in heat capacity during the phase transition of the $i^{\mathrm{th}}$ component. For TAGs, Wesdorp reports a rather small $\Delta c_{p,i}$ (Marangoni and Wesdorp 2012), moreover, the difference between $T_{m,i}$ and T is small, in the commonly investigated TAG temperature ranges, making the two heat capacity terms relatively small, compared with the enthalpy contribution, making them negligible and safe to cancel. Equation (7) is the commonly used simplified version:

(7) $$\ln\left(\frac{\gamma_{i}^{S}x_{i}^{S}}{\gamma_{i}^{L}x_{i}^{L}}\right)=\frac{\Delta H_{m,i}}{R}\left(\frac{1}{T}-\frac{1}{T_{m,i}}\right).$$

The Gibbs free energy of a specific phase can be obtained by considering the number of moles n of each $i^{\mathrm{th}}$ TAG component, where N is the total number of components in the mixture (Equation 8):

(8) $$G=\sum_{i=1}^{N}n_{i}\mu_{i}=G^{\mathrm{ideal}}+RT\sum_{i=1}^{N}n_{i}\ln\gamma_{i},$$

When the components in the system do not mix ideally, an excess Gibbs free energy contribution $g^{E}$ must be taken into account.

(9) $$g^{E}=G-G^{\mathrm{ideal}}=RT\sum_{i=1}^{N}n_{i}\ln\gamma_{i}.$$

$g^{E}$ can be calculated by using several models; one of the most common is the Margules one. Equation (10) describes the 2‐suffix Margules model, where the excess Gibbs free energy is calculated as a function of the interaction parameters $A_{ij}$ between two i and j TAGs:

(10) $$g^{E}=\sum_{i=1}^{N}\sum_{j=i+1}^{N}A_{ij}x_{i}x_{j}.$$

If the interaction parameters between two components are not symmetrical ($A_{ij}\neq A_{ji}$) then the more complex 3‐suffix Margules equation must be used (Equation 11):

(11) $$g^{E}=\sum_{i=1}^{N}\sum_{j=i+1}^{N}\left(A_{ij}\frac{x_{j}}{x_{i}+x_{j}}+A_{ji}\frac{x_{i}}{x_{i}+x_{j}}\right)x_{i}x_{j}.$$

In a multicomponent system, the application of the Margules equation assumes that a TAG pair contribution is the same in a binary and in a multicomponent system for the same binary composition.

The activity coefficient $\gamma_{i}$ can be finally obtained with Equation (12):

(12) $$RTln\gamma_{i}=\frac{g^{E}}{n_{i}}.$$

Due to the lack of experimental data for the melting enthalpy and temperature of pure TAG components, Wesdorp (Marangoni and Wesdorp, 2012; Wesdorp, 1990) developed a simplified, semiempirical model to estimate these two parameters from basic TAG information such as: fatty acids length, position, unsaturation, and the relative difference in the FA chain lengths in position sn‐1 or 3 and position sn‐2.

To address the same lack of experimental data, Zéberg‐Mikkelsen and Stenby (1999) developed an empirical group contribution method to calculate pure TAG melting temperature and enthalpy. TAGs were grouped based on the FA symmetry on the glycerol backbone. Experimental melting temperature and enthalpy values were used to fit the model parameters.

The Wesdorp model for pure TAG thermal properties was found to be predictive, useful, and accessible, and it was implemented in an open‐access web application named triglyceride property calculator (TPC) (Moorthy et al. 2017), which was recently improved (Seilert et al. 2021).

Seilert noticed that some of the data used in the original Wesdorp model were not consistent, as less thermodynamically stable polymorphs had a higher melting temperature than the more stable ones. These data were discarded from the upgraded TPC and were updated with more recent literature. This reparametrized model was found to be an improvement of the original one, being predictive for saturated TAGs but still not sufficiently predictive for unsaturated TAGs.

A more recent empirical model developed by Seilert and Flöter (2021) uses structural data to predict pure TAG melting enthalpy and temperature. The enthalpy is calculated as a sum of three contributions related to the glycerol backbone configuration, the methyl ending plane (which contains the FA chain length), and the lateral interactions between FA chains (which depend on polymorphism). This model is as accurate as the Wesdorp one and uses fewer parameters. They also gathered a more structure‐oriented database, which contained experimental data collected with both Differential Scanning Calorimetry (DSC) and X‐ray scattering techniques. The glycerol configuration and type of stacking were considered. Thermodynamic data are reported for each polymorph of a TAG, both in 2L and 3L configurations. The authors reported less data for unsaturated TAGs compared with fully saturated ones, and overall, lower enthalpy values compared with the melting points.

To describe the nonideal mixing behavior of TAG mixtures, activity coefficients are needed to calculate the nonideal contribution to the free Gibbs energy. Marangoni and Wesdorp (2012) and Wesdorp (1990) developed a way to calculate the interaction parameters based on experimental data. The model is based on the following assumptions: (1) TAGs in the α modification, due to their liquid‐like behavior, are considered to mix ideally (γ = 1); (2) the β′ and the β phase are considered to behave non ideally, and the activity coefficients are usually calculated using the Margules model. Additionally, the authors state that the two‐suffix Margules model is able to replicate simple systems and is unlikely to be valid in a real TAGs mixture, and that the three‐suffix Margules model, although more complex, can better describe the nonideality of TAG mixtures. Interaction parameters were obtained by fitting experimental phase diagrams obtained via DSC. Obtaining a phase diagram with DSC requires long measurements and could lead to imprecise results. To address this, Wesdorp proposes the addition of a third liquid TAG in the range of measurements, to (1) improve and speed up equilibration, (2) facilitate and accelerate metastable polymorphic transitions, and thus (3) obtain a more reliable melting point without kinetic effects. In addition to this, instead of constructing a phase diagram by selecting a few points from the DSC curves, the interaction parameters are fitted to the full set of DSC curves, whose peak shapes change depending on TAG interaction.

The Wesdorp model for mixtures has been widely used to predict fat mixture properties. In several works published by Teles dos Santos and colleagues, the Wesdorp approach was applied to several fat mixtures. Additionally, a method to calculate the TAG profiles of a fat mixture based on the FA composition only is reported. As the FA composition is easier to obtain experimentally compared with the TAG profile, the authors developed a combinatorial analysis approach, based on random distributions of the FAs on a glycerol backbone to generate the most likely TAGs profile (Teles dos Santos et al. 2014; Teles dos Santos et al. 2018, 2016; Hjorth et al. 2015a, 2015b).

Los and Flöter (1999) proposed a kinetic addition to Wesdorp's thermodynamic model. Since TAGs diffuse very slowly in the solid phase, the actual phase behavior could be very different from the thermodynamic one. In this model, the liquidus line is shifted by a fixed undercooling temperature while the solidus line is recalculated through their kinetic model based on linear growth on rough surfaces. Different kinetic constants and different degrees of supercooling are compared, showing the effect of kinetics on a purely thermodynamic diagram.

A different approach was used by Brinkmann and coworkers (Brinkmann et al. 2019), which used PC‐SAFT (perturbed‐chain statistical associating fluid theory) to predict pure TAGs and TAG mixtures properties. PC‐SAFT is a model based on the calculation of the residual Helmholtz energy, which is correlated to vapor pressure, liquid density, and activity coefficient. The simulated solid–liquid equilibrium of various TAG mixtures agreed with the experimental ones.

Pereira and coworkers (Pereira et al. 2019) used a UNIFAC model (UNIQUAC Functional‐group activity coefficient) instead of the Margules approach to address the nonideality of fat mixtures, both in the liquid and solid states. The SFC curves of complex TAG mixtures were calculated. In some cases, the SFC trend was predicted successfully, but the majority of the calculations underestimated the solid fraction at low temperatures. The authors demonstrated that nonideality of the liquid phase must be considered when describing complex mixtures of TAGs.

In a recent article (Marangoni et al. 2023), a new model for the SFC calculation of cocoa butter and selected similar fat mixtures, made by one fixed polymorph solid phase and one ideal liquid, is reported. Pure melting TAG data are calculated with the Wesdorp pure TAG model (Marangoni and Wesdorp 2012) implemented in the TPC (Moorthy et al. 2017). Instead of evaluating pairwise TAG binary interactions, solid state nonideality is tackled by considering a single TAG solid state activity coefficient, which results from the interaction of a single TAG with a solid medium constituted of all other TAGs. A cooperativity melting index is included as well, representing the TAG connectivity in the crystal. Although the authors underline that further work is needed to turn this model into a predictive one, they showed a good qualitative fit of their nonlinear SFC model to experimental SFC points of 3 TAG mixtures, while higher, up to 5 TAG mixtures, were more sensitive to initial conditions.

More recently, Schaink (2023) used a simplified approach to the SFC calculation, considering a complex, nonideal mixture of TAGs as the equivalent of the sum of all possible combinations of binary TAG mixtures dispersed in an inert solvent. Based on these assumptions, the solidus points were calculated for every possible binary combination through the Hildebrand equation (Equation 13).

The Hildebrand equation is the simplified version of Equation (7): ideal mixing is assumed for the liquid phase ($\gamma_{i}^{L}=1)$, and the solid phase is assumed to be composed of immiscible pure components ($\gamma_{i}^{S}\;x_{i}^{S}=1$) (Maximo et al. 2014).

(13) $$\ln x_{i}=\frac{\Delta H_{m}}{R}\left(\frac{1}{T_{m}}-\frac{1}{T}\right).$$

A series of SFC points is then calculated by summing all the solidus obtained by TAG pairs, weighted by their molar fractions in the temperature range. Although this method presents some limitations, such as considering only one polymorph at a time, it takes into consideration even low concentration triglycerides that are shown to impact the crystallization behavior. Schaink applies this approach to describe the SFC of cocoa butter, palm oils, and palm kernel oils (from 9 to 19 TAGs), showing a good qualitative agreement, considering the simplified model assumptions.

### 2.3 Experiments for Model Parametrization

As shown in the previous section, melting enthalpy and temperature of pure TAGs together with a model for nonideal interactions are needed to develop a thermodynamic model to describe the SFC of fat mixtures. Hence, specific experimental techniques are necessary to gather thermodynamic data for pure components, binary mixtures and to validate the developed models for more complex mixtures. It is worth noticing that ensuring TAG purity is essential for the precise estimation of thermodynamic parameters; impurities (e.g., other TAGs, minor lipid components) can significantly affect the melting temperature and enthalpy. Hence, a preliminary assessment of purity, for example via chromatographic measurement, is recommended.

DSC is the most widely used technique to measure melting temperature and enthalpy of pure components and to estimate the activity coefficients of binary TAG mixtures. This technique does not provide structural or chemical information on the phases measured; hence, the measurement of thermal properties of metastable polymorphs or the estimation of the weight ratio of different solid phases in a binary mixture can be challenging. The use of nuclear magnetic resonance (NMR) for the estimation of the SFC of mixtures of TAGs presents the same limitations as DSC. X‐ray scattering measurements at variable, controlled temperature can provide structural information during the estimation of the thermal properties required for thermodynamic models; this can improve the accuracy of the data collected and the developed thermodynamic model. However, for complex TAG mixtures, this technique still does not provide information on the TAG composition of each polymorph.

A systematic effort in measuring pure TAG data was reported in Marangoni & Wesdorp (Marangoni and Wesdorp 2012; Wesdorp 1990) in which simple experimental procedures were set up to obtain melting temperatures and enthalpies of α, β′, and β polymorphs of various TAGs.

The majority of pure TAGs’ thermodynamic data were collected by DSC. Data regarding the β polymorph were obtained by the melting curves of TAG samples, after keeping them at room temperature for months to years. On the other side, an α metastable polymorph was obtained by rapidly quenching the liquid TAG. To obtain the pure β′ polymorph, two separate strategies were tested: either slowly heating until the start of the melting of the α modification and then set the system at that temperature while the melting curve was recorded; or, for the second strategy which was used in case a β′ to β transition happened during the previous procedure, the TAG system would be fully melted and rapidly quenched to 1 or 2°C above the melting point of the α polymorph. Despite this, the authors report the presence of phase interference, thus indicating the difficulty of obtaining and measuring pure polymorphs with DSC.

The revisited model by Seilert et al. (2021) was based on a pure TAG database, while thermodynamic constraints were applied to initial data and to the parameter estimation procedure, in the sense that data regarding polymorphs of the same TAG not following the stability order of melting points ($\;T_{m,\alpha }<T_{m,\beta^{\prime }}<T_{m,\beta }$ ) were excluded. Experimental data from techniques other than DSC were included. Small‐angle and wide‐angle X‐ray scattering (SAXS and WAXS, respectively) experiments performed at controlled temperature (e.g., using a temperature‐controlled stage or sample holder) were used (Takeuchi et al. 2003; Ueno et al. 1997) as these techniques allowed for a clear identification of the temperature of specific polymorph crystallization and melting. Different reflection order SAXS peaks relative to the same phase are equally spaced, and this distance can be correlated with the long d‐spacing. This value can be used to obtain information on the lamellar stacking (2L, 3L, etc.). WAXS peaks are correlated to the short d‐spacing, which is informative of the distance between FA chains in the TAG sub‐cell, thus revealing the specific polymorph (Figure 1).

Other works (Bayés‐García et al. 2013; Minato et al. 1997; Nakanishi et al. 2018; Takeuchi et al. 2002) report both SAXS/WAXS experiments and DSC. These works show that, by keeping the same thermal treatment, it is possible to correctly identify the polymorph while obtaining enthalpy values.

Regarding the nonideality of fat mixtures, Wesdorp (Marangoni and Wesdorp 2012; Wesdorp 1990) collected binary TAG mixtures data to obtain the activity coefficients needed for an accurate description of the nonideal behavior in a more complex sample. The excess Gibbs energy of the α polymorph is evaluated by comparing NMR experimental data and calculated melting ranges, concluding that mixing in the α modification can be considered ideal. For the β′ and β polymorphs, the mixing behavior is nonideal, and Margules parameters needed to obtain the activity coefficients are estimated experimentally. In the work from Marangoni and Wesdorp (2012), two experimental approaches are reported. The first one was to obtain phase diagrams of binary TAG mixtures by performing DSC heating and cooling ramps for different component fractions. The second approach consisted of obtaining the DSC crystallization curve of ternary mixtures consisting of the two TAGs whose interaction parameter is needed, with a surplus of a third liquid TAG immiscible with both components of the binary mixture (e.g., triolein for binary mixtures of tri‐saturated TAGs). According to the authors, the presence of a third liquid component grants a better and faster stabilization of the studied binary TAGs mixture at the tested temperatures. In fact, the presence of an immiscible liquid solvent speeds up the mass transfer processes involved in polymorphic transformation into the stable forms and phase separation. It is worth noticing that the presence of a solvent can lower the melting temperatures of the binary mixtures. DSC curves obtained experimentally were then fitted using the 3‐suffix Margules Model. The crystallization of binary TAG mixtures in the liquid medium presented two peaks on the DSC curve; the interaction parameters $A_{ij}$ and $A_{ji}$, of the 3‐suffix Margules equation influences the shape of the first peak and the temperature difference between the two peaks.

Pereira et al. (2019) used SFC measurements to obtain the parameters needed to fit a predictive UNIQUAC model to address the nonideality of some natural fat mixtures.

In the model proposed by Marangoni et al. (2023), X‐ray diffraction techniques were performed to determine the “apparent average polymorphism of the system”, since the solid state is assumed to crystallize in a single polymorph, in the modelled TAGs mixture. This information was then used to select the melting point and enthalpies of each TAG polymorph detected for the calculation of the SFC curve. This approach strongly relies on experimental data for the correct fitting of SFC, and the solution is not numerically stable when more than three TAGs are considered in the mixture.

Finally, it is worth reminding that the FA profile of a TAGs mixture can be obtained by gas chromatography (GC) (Teles dos Santos et al. 2016), while the TAG profile can be obtained by high‐pressure liquid chromatography (HPLC) (Marangoni et al. 2023). This last information is essential as the relative position of the FA chains attached to the glycerol backbone strongly affects polymorphism and hence, solid‐state properties.

### 2.4 Gaps and Limitations of Current Thermodynamic Models

Thermodynamic modeling of TAG mixtures is an interesting subject for research; models are constantly evolving and improving, while incorporating new and more robust data or knowledge. Despite this, some gaps can still be underlined, both from an experimental and modeling point of view.

A considerable number of natural fats contain unsaturated TAGs (e.g., tri‐unsaturated ones such as triolein, but also di‐unsaturated TAGs such as OOP and OOS); despite this, there are scarce data on pure unsaturated TAGs reported in literature, a lot less compared with fully saturated ones (Seilert and Flöter 2021). Hence, a proposed future direction to properly study the crystallization of natural fat mixtures is to put more focus on characterizing unsaturated triglycerides. From the literature analysis reported by Seilert et al. (2021), it is also possible to notice fewer data on β′ polymorphs compared with the more stable β forms. Reliable pure TAG data for all possible polymorphs is crucial for any thermodynamic description of fats, and simple models for pure TAG properties prediction have been reported (Moorthy et al. 2017; Seilert and Flöter 2021) and are a useful computational tool to integrate experimental databases. Nevertheless, more data on unstable polymorphs should be collected.

The vast majority of experimental data for thermodynamic model parametrization was collected using DSC: this thermal analysis is a fast and commonly used technique for melting and crystallization temperatures and enthalpies determination. However, this characterization technique presents some drawbacks; the thermal peak area, intensity, and position are influenced by the experimental conditions, in particular by cooling and heating rates applied.

Moreover, DSC does not provide any structural information (e.g., number, stacking, and subcell type) regarding the polymorphs present during the experiments and the different melting and crystallization events occurring during the analysis (e.g., fitting of overlapping endothermic and exothermic events is extremely complex without knowing the relative amount of the polymorphs present). Polymorph identification is possible by comparing melting and crystallization points only, which is particularly difficult in the case of complex mixtures due to overlapping peaks of either multiple polymorphic forms or solid phases of the same polymorphic form but different TAGs compositions (e.g., due to immiscible TAGs in the mixture). Additionally, DSC is not ideal for the measurement of metastable polymorphs (Seilert and Flöter 2021), as polymorphic conversions might happen during the measurement without being clearly detected, due to the possible overlapping of endothermic and exothermic peaks. This kind of polymorphic structural information can only be obtained with X‐ray scattering techniques. An experimental setup, which couples X‐ray diffraction and DSC, was reported by Ollivon et al. (2006), which allows for simultaneous structural and thermal characterization of fat mixtures. To develop better thermodynamic models, more structural data should be collected together with thermal profiles (e.g., using variable temperature X‐ray diffraction setups). It is worth noticing that this experimental approach still lacks detailed information on the TAGs composition of each detected polymorph (even if some simplified assumptions can be made from SAXS data).

A promising contribution to pure TAG melting points prediction, based on a machine learning approach, has been published recently (van Herck et al. 2024). This work is based on fine‐tuned large language models (LLMs), which were able, starting from a TAG melting point literature dataset, to correctly predict if a given TAG melting temperature would be higher or lower than the median temperature of the dataset. This approach can be a potential starting point for the application of machine learning techniques in predicting lipid properties.

The thermodynamic Wesdorp model for mixtures was applied to predict the SFC of natural fats. The general trend is reproduced correctly compared with experimental data, but a precise curve prediction has not been achieved yet. In some cases, the polymorph is not specified (Teles dos Santos et al. 2013), and for some systems, the difference between simulated and experimental data is significant. Often, the thermodynamic models of the solid fat content assume that all the TAGs in the mixture crystallize in the same polymorph (Brinkmann et al. 2019; Teles dos Santos et al. 2016); this is an unrealistic approximation, and the simulated profile is not always accurate. More effort should be put into developing models that can deal with multiple polymorphs.

Large differences between simulated and experimental SFC curves could also be attributed to the simplified TAG profile of the modelled mixtures, as components in low concentrations are not considered in calculations but could, nevertheless, confer a significant impact on crystallization. In a work by Teles dos Santos and coworkers (Teles dos Santos et al. 2013), only TAGs accounting for 85% in mass are considered, while Marangoni et al. (2023) considered only the three main TAGs of cocoa butter to describe this mixture, representing also approximately 85% of the total TAG composition. While these assumptions can simplify the specific reported systems, the generalization to other fats could lead to great differences between experiments and models.

The minority compounds are only considered by Schaink (2023), with the drawback of predicting separate SFC curves for each polymorph and assuming ideal mixing, and while the β′ and the β polymorph curves were closer to the experimental ones, the α curve underestimated the solid percentage at the given temperatures.

Regarding fat mixtures, their nonideal behavior is often subject to major approximations; for example, the liquid phase is often considered to be ideal. Marangoni & Wesdorp (2012) state that this holds true only if the difference between the FA chains’ length of the TAGs forming the mixture is not larger than 15–20 carbon atoms. The DSC performed on binary TAG mixtures in a liquid TAG medium was used to obtain the activity coefficients to describe the nonideal behavior of the β′ and the β polymorphs; nevertheless, limitations were found with this method. The criticalities of DSC in determining polymorphism still apply, and using a surplus of a third liquid TAG to obtain binary interaction parameters could influence the thermogram due to miscibility issues.

For nonideality of the solid phase, the most common model used is the Margules one (Marangoni and Wesdorp 2012; Wesdorp 1990). However, this is not the only available way to address nonideality. Predictive UNIQUAC (UNIversal QUAsi‐Chemical) and UNIFAC (UNIQUAC Functional‐group Activity Coefficients) provide an alternative approach to obtain activity coefficients for TAG mixtures, both in multicomponent (Pereira et al. 2019) and binary systems (Pereira et al. 2022). UNIQUAC describes the nonideality by size, shape, and energy interactions using fitted binary interaction parameters; its predictive variant does so by only estimating from pure molecular data only, which was used to estimate the activity coefficient in the solid phase. UNIFAC keeps the same idea as UNIQUAC, but through a group contribution method; this strategy was used instead to estimate the activity coefficient in the liquid TAG. The detailed mathematical description of UNIFAC and UNIQUAC methods is reported in the original papers by Larsen et al. (1987) and Coutinho (1998), respectively. Andrade and coworkers (Andrade et al. 2023), studied solid–liquid equilibria of vitamin E and TAG systems. In this work, COSMO‐SAC (COnductor‐like Screening MOdel—Segment Activity Coefficient), along with Margules and UNIFAC, were used to predict the activity coefficient. COSMO‐SAC is a segment model (similar to a group contribution method) derived from COSMO‐RS. COSMO‐RS uses σ‐profiles (surface screening charge densities) from so‐called COSMO calculations to describe molecular segment interactions. For further mathematical details on COSMO models, we recommend that readers check the original works of Klamt for COSMO‐RS (Klamt 1995; Klamt et al. 1998) and the SAC formulation from the study by Lin and Sandler (2002).

This approach represents one of several possible strategies for describing the nonideality of fat mixtures. Used as an alternative to, or in combination with, the widely adopted Margules model, it could lead to more accurate thermodynamic calculations that better reflect the nonideality of real fat systems.

## 3 Kinetics Models to Describe Crystallization of Fat Mixtures

### 3.1 Fat Crystallization Kinetics Introduction

The previous section dealt with thermodynamic models to describe TAG crystallization. While thermodynamics provides essential information on the solid–liquid equilibrium state and phase miscibility of TAG mixtures, no accurate crystallization description would be complete without considering the kinetics of growth, nucleation, and polymorphic transformations. These factors influencing fat crystallization should be considered in both product and process design to ensure that the final food product has the desired functionality and macroscopic properties.

Polymorphic transitions in TAG mixtures are not always fast, and the transformation to the most stable polymorph can take even weeks or months. The presence or absence of a specific polymorph and the coexistence of different polymorphs in a crystallizing mixture are greatly influenced by the crystallization conditions, such as the application of specific thermal protocols or the presence of shear, but also the presence of other substances and impurities. There is a lack of models that address the complex kinetics of the polymorphic behavior of fats; this could also be due to the fact that not all the experimental techniques used in lipid characterization can accurately detect polymorphic transformations.

Aside from polymorphic transformations, kinetic data are fundamental in process control and design. A deep knowledge of crystallization kinetics offers great value in rationalizing product design; investigating how key parameters such as induction time, nucleation, crystal growth rates, and how they are affected by temperature profiles (isothermal or cooling) or application of shearing could lead to a deeper understanding of fat crystallization phenomena. To do so, an accurate characterization of the crystallization process and robust models are needed to better describe the overall TAG crystallization phenomena.

This is particularly relevant for TAGs, being relatively large molecules, they diffuse slowly, especially when the melt starts to solidify, and kinetics is the predominant factor in mixture properties. An effect of this in TAG mixtures is represented by composition gradients even in the presence of mixing: nuclei of higher melting TAGs crystals tend to form first upon cooling, then the other lower melting TAGs would crystallize around these nuclei. The slow diffusion rate of TAG molecules during crystallization can lead to a compositional inhomogeneity within the solid mixture (Garti and Satō 2001; Walstra 2002).

To precisely estimate TAG crystallization kinetics, nucleation, and growth should be measured separately. While it is not possible to practically separate these crystallization phenomena, there are ways to selectively measure them, for example, using induction time measurement for primary nucleation kinetic estimation or optical imaging to quantitatively estimate crystal growth. Having said that, measuring crystallization phenomena is not trivial for TAG mixtures, due to the detection limits of commonly used instruments in measuring the exact time of nuclei formation, and due to the irregularity and the aggregative behavior of TAGs crystal growth. In general, measuring induction time is a common way to estimate nucleation kinetics (Himawan et al. 2006). It must be noted that the measured induction time also contains the time necessary for the crystal to reach a size actually detectable by the instrument used for the measurement; consequently, different measurement techniques provide different induction times (Wright et al. 2000).

### 3.2 Common Kinetic Models to Describe TAG Crystallization

This section describes the most widely used models to describe TAGs crystallization. Each model has its own advantages and disadvantages, based on formulation strategy (empirical vs. first‐principle models), fitting accuracy, and computational cost. The choice of one model over another depends on the needs of the users (e.g., fast measurement vs. accuracy).

The most common model for the description of isothermal crystallization processes is the Avrami model (Avrami 1939, 1940). It was originally developed to describe nucleation and growth of metal alloys, and then it was applied to polymers and to fat crystallization. The Avrami model was developed for systems that crystallize with a constant growth rate and morphology; these assumptions are not always true for TAG systems since their growth rate and morphology may not be constant. Moreover, polymorphism cannot be described in the original Avrami formulation without any modification (Foubert et al. 2003; Himawan et al. 2006). Despite its empirical nature, this model is still widely used due to its simplicity and low computational costs, which makes it convenient, for example, for process control where speed of sampling is more critical than accuracy.

Nucleation, expressed as the number of nuclei per unit volume, is assumed to be either sporadic or instantaneous. In sporadic nucleation (also known as progressive nucleation), the number of nuclei linearly grows with time, while in instantaneous nucleation, most of the nuclei are formed at the beginning of the solidification process. This second case is often used to describe a seeded crystallization. The growth is assumed constant (Foubert et al. 2003; Himawan et al. 2006). The solid fraction is expressed as Equation (14):

(14) $$X_{\mathrm{solid}}=1-e^{-kt^{m}},$$

where k depends on the type of nucleation and the growth rate, and m depends on the nucleation type (sporadic or instantaneous) and growth dimensionality (rod‐like, plate‐like, spherical crystals). The m exponent should be an integer, but the fitting to experiments yields fractional values. This can be attributed to variation in nucleation and growth rates or changes in dimensionality during growth.

A modified version of this model, known as “modified Avrami” or “Avrami‐Erofeev”, was proposed by Khanna and Taylor (1988). This modified Avrami model is a reparameterization of the original one, in which k is replaced by $k^{\prime }$ Equation (15):

(15) $$X_{solid}=1-e^{-{\left(k^{\prime }t\right)}^{m}},$$

where k’ is Equation (16):

(16) $$k^{\prime }=k^{\frac{1}{m}}.$$

This reparameterization transforms k from a $m^{\mathrm{th}}$ order constant to a first order one. The authors state that this reparameterization better describes the crystallization rate. The lack of a clear theoretical basis for this reformulation of the Avrami model has limited its use (Marangoni 1998).

The Gompertz model (Gompertz 1825) was initially applied for bacterial growth (Zwietering et al. 1990) and later used by Kloek et al. (2000) to describe isothermal fat crystallization. The general equation of the model, which describes the solid fraction over time, is represented as Equation (17):

(17) $$s_{i}\left(t\right)=S_{\max,i}\cdot \exp\left\{-\exp\left[\frac{\mu_{i}e}{S_{\max,i}}\left(\lambda_{i}-t\right)+1\right]\right\}.$$

For each separate i polymorph, we can define $S_{\max,i}$ as the maximum fraction of solid fat, $\mu_{i}$ is the maximum specific growth rate during the crystallization and $\lambda_{i}$ is the induction time, all of them considered for the i−th polymorph. This model can be adapted to cases in which multiple simultaneous polymorphs crystallize by simply expressing $s_{tot}\left(t\right)$ as the sum of solid contributions from $s_{i}\left(t\right)$ for each i polymorph (Marangoni 2018). This approach is valid as long as each parameter can be fitted separately from those of other polymorphs, requiring experimental techniques, such as X‐ray scattering (Kloek et al. 2000), that are able to detect and quantify single polymorph contribution to the crystallization.

The model proposed by Berg and Brimberg (1983) is based on an analogy between fat crystallization, flocculation, and aggregation of colloids. These two phenomena are described by Equations (18) and (19):

Aggregation:

(18) $$C-C_{0}=-k_{n}{\left(t-t_{0}\right)}^{n}.$$

Flocculation:

(19) $$\ln\frac{C}{C_{0}}=-k_{n}{\left(t-t_{0}\right)}^{n}.$$

Foubert et al. (2002) developed a more versatile model representing the crystallization process as a combination of a forward first‐order reaction and a $n^{\mathrm{th}}$‐order reverse reaction. The forward reaction represents the crystal formation, while the reverse reaction is caused by the remelting of crystals due to heat dissipation phenomena during crystallization. The crystallization kinetics can be written as Equation (20):

(20) $$\frac{dh}{dt}=K_{n}\cdot h^{n}-K_{1}\cdot h,$$

where h is the fraction of crystallizable fat, which decreases when the crystallized fat fraction increases, t is time, and $K_{n}$ and $K_{1}$ are, respectively, the rate constant of the $n^{th}$ order reverse reaction and the rate of the first‐order forward reaction. This model was shown to better fit, compared with Avrami and Gompertz models, isothermal crystallization curves of cocoa butter and milk fat samples, obtained with DSC and NMR. In a later work from the same group, this model was extended to two‐step crystallization cases (Foubert et al. 2006). A major assumption of this model is that the polymorph nucleates first and then transforms to β′; this polymorph is assumed not to directly form from the melt. However, in the case of fat systems where there β′ is directly nucleated from the melt, the Foubert model would need to be adapted (Dewettinck et al. 2004).

Hjorth et al. (2015a) used a different approach to model the kinetics of fat crystallization. Primary nucleation was described using the classical nucleation theory, which expresses the nucleation rate as Equation (21):

(21) $$J=J_{0}\exp\left(\frac{-\Delta G}{k_{B}T}\right),$$

where J and $J_{0}$ are the nucleation rate and the maximum nucleation rate, respectively, $k_{B}$ is the Boltzmann constant, and *T* is the temperature, while the activation Gibbs free energy ΔG can be rewritten as Equation (22):

(22) $$\Delta G=\frac{-16\pi \sigma^{3}V_{s}^{2}}{3k_{B}^{2}T^{2}{\left(\ln\frac{C}{C^{*}}\right)}^{2}},$$

where σ is the surface tension, $V_{s}$ is the molecular volume and C and $C^{*}$ are the molar concentration of a TAG in a mixture and its equilibrium concentrations, respectively.

Then, crystal growth was modeled by Hjorth et al. (2015a,b)using a modified Burton Cabrera Frank model (Equation 23).

(23) $$G_{\mathrm{vol}}=K_{G}A^{Pa}\Delta C,$$

where $G_{\mathrm{vol}}$ is the volumetric crystal growth rate, $K_{G}$ is the growth constant, A is the surface area of the crystals, $P_{a}$ is a factor that takes into account the possible nonavailability of surface sites due to the presence of other crystals, thus slowing the growth rate, while ΔC is the difference between the actual molar TAG concentration in the liquid phase and its equilibrium concentration at the operating temperature.

**FIGURE 3:**
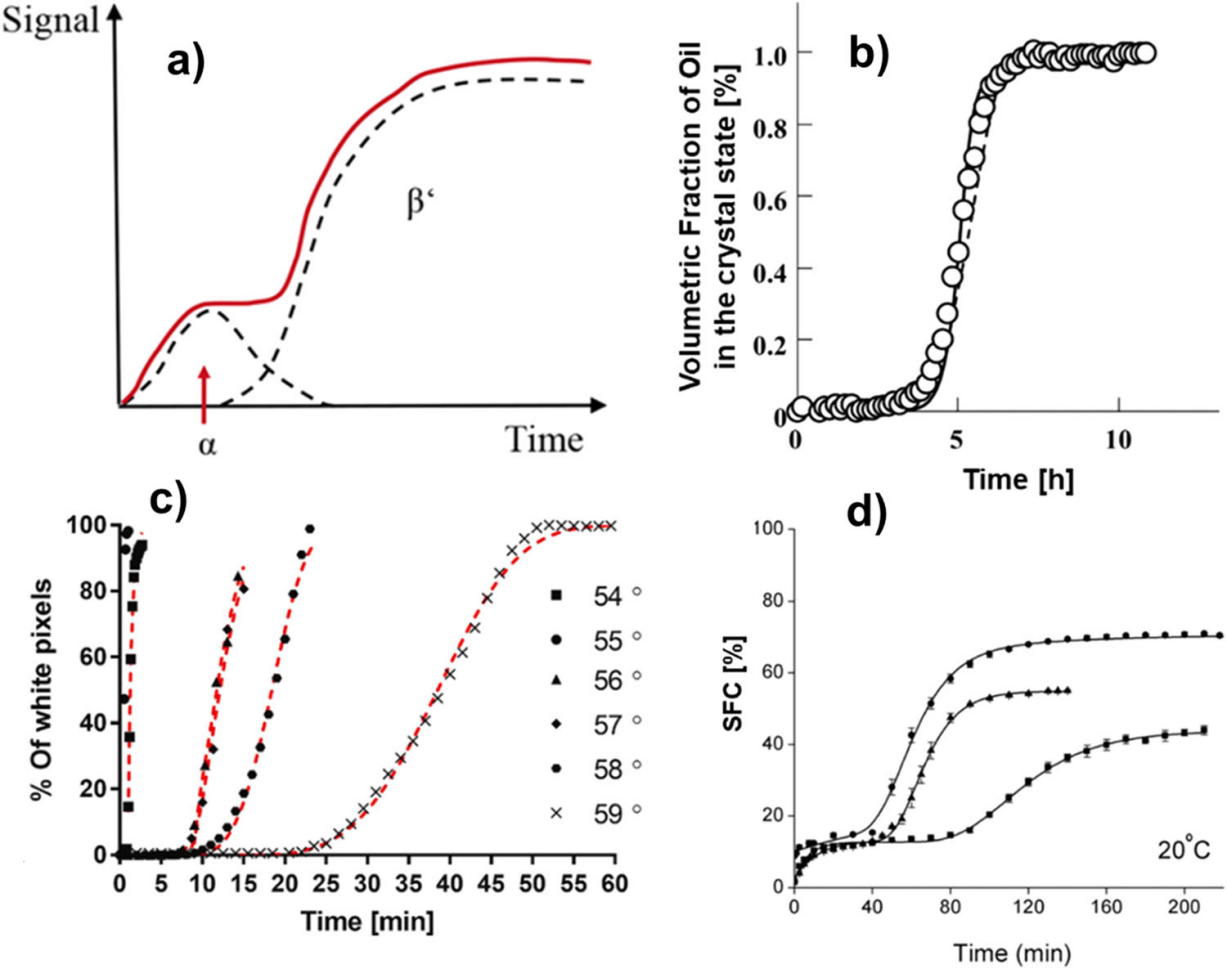
(a) Generic example of an experimental crystallization curve during a polymorph transformation (red). Dotted lines indicate a single polymorphic contribution to the curve. *Source*: Adapted from Seilert et al. (2024). (b) Fitting of experimental volumetric fraction measurements (dots) with the Avrami model (dashed curve), adapted from (Miyagawa et al. 2018). (c) isothermal crystallization of a TAG at different temperatures. Experimental points are counts of total white pixels obtained through PLM fitted with the Avrami model (red dashed curve). *Source*: Adapted from (Golodnizky and Davidovich‐Pinhas 2022). (d) isothermal two‐step crystallization of different cocoa butter samples measured with pNMR (symbols) fitted with two‐step Gompertz model (solid line). *Source*: Adapted from Bootello et al. (2013).

Miyagawa et al. (2018) proposed a new model to describe the isothermal crystallization of rapeseed oil as an autocatalytic chemical reaction. The liquid was assumed to be able to transform irreversibly into a crystal and reversibly into a metastable, highly concentrated liquid state (e.g., prenucleation clusters), which favors the formation of crystal nuclei. This highly concentrated liquid state can transform back to the liquid state or irreversibly to a crystal. Although this model performed better compared with Avrami in fitting experimental data, it presents some limitations. Fitting is based on time‐resolved dilatometry measurements (Figure 3b), which may not be common in lipid‐specialized laboratories. The model also fits volumetric parameters, only ignoring TAG composition and polymorphs, and lastly, it was validated only for a single case of rapeseed oil under isothermal storage, limiting the model's applicability in other cases.

### 3.3 Experimental Model Validation

Several experimental techniques have been used to study fat crystallization both during cooling and in isothermal conditions. It is worth noticing that each of the techniques that will be described in this section works at a specific length scale; for example, optical imaging enables the study of micrometric objects, whereas wide‐angle X‐ray scattering can probe interatomic distances. Hence, a direct comparison among techniques is not possible, and we will only discuss the type of information and the practical advantages/disadvantages of each analytical technique in relation to the study of fat crystallization. Instrumental cost, sample preparation, speed of measurement, and ease of data interpretation are considered to compare each technique. Nevertheless, we would like to point out that sensitivity and data quality are essential for the development of reliable thermodynamic models.

One of the most straightforward methodologies to determine the rate of crystallization is using turbidity measurements. By measuring the intensity of transmitted light through a crystallizing sample, it is possible to obtain an estimate of the amount of solid formed and the rate of its formation. This technique was employed by Dibildox‐Alvarado and Toro‐Vazquez (1998) and Toro‐Vazquez and Dibildox‐Alvarado (1997) applied to the crystallization of tristearin in sesame oil. The experimental data were then fitted using the “modified Avrami” model. Miyagawa et al. (2018) monitored the crystallization behavior of rapeseed oil by measuring the volume change using dilatometry and turbidity, to obtain crystallization curves that were fit with their newly developed model.

X‐ray diffraction is a powerful technique that allows nano‐ and sub‐nanostructural information necessary for the correct polymorph identification during crystallization. In particular, Synchrotron radiation SAXS and WAXS experiments provide fast and high‐resolution diffraction patterns also during thermal profiles and using specific setups (e.g., rheoSAXS), making this technique the ideal one to study crystallization kinetics. Synchrotron facilities can be accessed by any academic research across the globe, and experimental proposals are assessed via peer review, making this technique relatively widely available. Nevertheless, good‐quality data can also be obtained using benchtop X‐ray scattering setups.

In fact, crystallization curves can be built by considering the total contribution of each specific polymorph to the total solid (Figure 3a). In some papers, a kinetic model fitting was made on crystallization curves obtained with X‐ray measurements (Dewettinck et al. 2004; Foubert et al. 2006; Ladd Parada et al. 2019; Mazzanti et al. 2009). These measurements have also shown prenucleation TAGs clustering phenomena (Ladd Parada et al. 2019) that suggest that the nucleation mechanism for fat mixtures is more complex than the one described by the classical nucleation theory. Indeed, some of the models described in the previous section should be revisited in view of these experimental findings.

There are also many experimental works based on the use of this technique (Arita‐Merino et al. 2020; Miyagawa 2021; Pratama et al. 2021; Seilert et al. 2024; Simone et al. 2024) to measure polymorphic transition kinetics.

Polarized light microscopy (PLM) is a rather simple and inexpensive technique that has been used to monitor fat crystallization in the micron‐size range (Figure 3c). Its main drawback is the low accuracy in the detection of the induction time due to the fact that fat crystals are not visible immediately upon nucleation but only after reaching the micrometric size (Golodnizky and Davidovich‐Pinhas 2022).

A rather simple experimental technique to indirectly estimate crystallization kinetics is rheology. By evaluating the shear force applied to a crystallizing TAGs mixture, it is possible to obtain a curve that depends on the concentration of solid. Bölük et al. (2024) applied this technique to study the crystallization of cocoa butter samples. The experimental curves were then fitted with a Gompertz model. Geary and Hartel (2017) fitted both the SFC curve and rheology measurements with the Avrami model.

Pulsed nuclear magnetic resonance (pNMR) is the standard technique for monitoring the evolution of SFC during crystallization. This technique was used by several authors; for instance, Szydłowska‐Czerniak et al. (2017) monitored the SFC of cocoa butter during an isothermal crystallization; the Foubert model was found to fit well the two‐step SFC curve. In another work by Bootello et al. (2013), pNMR was used to obtain the isothermal SFC curve of cocoa butter, and the crystallization mechanism was investigated with DSC and XRD. Experimental two‐step SFC curves (Figure 3d) were then fitted using an exponential model for the first rapid step and a Gompertz model for the second step. The Gompertz model can model different polymorph contributions, but since pNMR is not polymorphic‐specific, a hybrid, two‐step strategy was needed. The authors attributed the first step to the formation of α or β′ polymorph and the second step to the polymorphic transformation to β′ or β.

Aside from the standard pNMR procedure, SFC curves can be obtained with acoustic measurements. In a work by Povey and coworkers (Povey et al. 2009), ultrasound velocimetry was used to obtain SFC curves of a cocoa butter emulsion, while Metilli et al. (2022) used pulsed ultrasound and supervised machine learning to estimate the SFC during crystallization of cocoa butter oleogels. In this work, it was observed that this technique was not as effective in detecting metastable polymorphs and monitoring polymorphic transformations.

A less common technique for fat crystallization is Fourier Transform Infrared (FT‐IR) spectroscopy. In a work by Ioannidi et al. (2023), the spectroscopic measurement of chocolate crystallization rate is reported. Selected spectral regions were analyzed through a multivariate curve resolution‐alternating least squares method to distinguish the amorphous and crystalline contributions. The amount of crystalline material was then fitted with the Avrami model. Although IR analysis is a cheap, easy, and fast technique to track crystallization trends, this setup and the following fitting cannot distinguish overall crystalline or amorphous packing at the TAG level.

### 3.4 Gaps and Limitations in Validating Kinetics Models

TAGs crystallization kinetics, despite their importance, are difficult to estimate (Foubert et al. 2003; Himawan et al. 2006).

The simple and empirical Avrami model is one of the most used to quantify crystallization kinetics. Other models can fit experimental data better, but they are fully empirical as their parameters often lack a physical interpretation of the crystallization phenomena (Ioannidi et al. 2023).

Turbidity is often used to obtain an estimate of solid fat content during fat crystallization. This technique, while fast and cheap, has some drawbacks as heterogeneous nucleation and secondary crystallization may be overestimated due to changes in crystals birefringence over the crystallization process (Toro‐Vazquez et al. 2000). Moreover, Marangoni (1998) highlights the issues related to detector saturation, in the sense that the maximum turbidity measured often corresponds to the point where the sample becomes occluded by crystals, and not to the end of the crystallization process. For this reason, this technique is not suitable for the Avrami model, which requires the maximum possible value of SFC for the studied system. It is worth noticing that light scattering is strongly influenced by crystal size and shape, which could make turbidity an unreliable technique for the crystallized fat fraction determination.

DSC and pNMR are other commonly employed measuring techniques, but they also present some drawbacks. DSC could be useful to gather a general idea of the crystallization process, but not quantitative information on the type, number, and weight ratio of polymorphs present in the system. The reasons are the same as mentioned in Section 2, such as the different crystallization peak form, the position based on experimental conditions, and the lack of structural information. pNMR is a fundamental technique to gather SFC information and can be considered very reliable for determining the percentage of crystallized triglyceride. Recently, the possibility of adding shear to this technique (rheoNMR) has further improved its applicability (Rebry et al. 2021). Acoustic measurements applied to TAGs crystallization could also be a cheaper, faster, and online alternative for the determination of the SFC, although less accurate than pNMR (Metilli et al. 2022; Povey et al. 2009). X‐ray scattering experiments are a fundamental tool for a deeper understanding of the kinetic mechanism of polymorphic transitions in the crystallization process, although precise quantification when more polymorphs are present is still challenging.

## 4 Molecular Modeling of TAGs Crystallization

### 4.1 Introduction to Molecular Modeling

The previous sections were focused on modeling fat crystallization from a thermodynamic and a kinetic point of view. This section will focus on the advancements in molecular modeling strategies that have been used to describe TAGs crystallization. Molecular modeling could help to better understand the structure and the dynamics of crystallization at a smaller scale compared with the other presented approaches.

A valuable technique to investigate fat systems, in which weak atomic interactions are prevalent, is Molecular Dynamics (MD). This is based on a classical Newtonian evaluation of the forces of an atom (Equation 24):

(24) $$F=ma=m\frac{d^{2}r}{dt^{2}},$$

where F is force and m and a are mass and acceleration, respectively; the latter can also be expressed as the second derivative of the displacement r.

**FIGURE 4:**
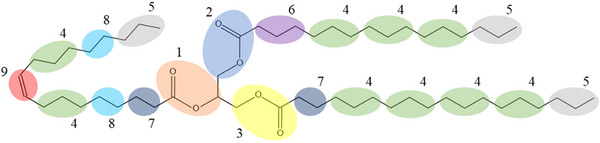
Nine bead coarse grained (CG) force field mapping of an unsaturated TAG. Each color and number represent a different parametrized bead. *Source*: Adapted from Cordina et al. (2024).

With this type of simulation, it is possible to evaluate the displacement and the velocity of all the atoms in a system. This simulation technique is effective when the time between two consecutive simulations (time step) is very small (1 or 2 fs), since the force magnitude needs to be constant during the atom displacement (Euston 2013). Due to the relatively large number of atoms that constitute TAG molecules, an atomistic simulation would be computationally intensive (Brasiello et al. 2012). A common way to carry out simulations with these kinds of molecules is to use coarser models. In Coarse Grained Molecular Dynamics (CG‐MD) simulations, instead of simulating every single atom, larger pseudo‐atoms (commonly called beads) are considered, as reported in Figure 4. Atoms with similar physicochemical properties are grouped in the same bead (e.g., ester group, carbon‐carbon double bonds, middle chain groups, or ending chain groups); this allows for a lower computational cost, and bigger timesteps (tens of fs) but a loss of information during the simulation at the atomic level. The CG‐MD approaches are among the most promising techniques to model liquid–solid transitions of TAGs (Euston 2013).

Newtonian forces, and consequently motion, acting on particles (atoms or beads) in a simulated system can be described by a force field U (Equation 25):

(25) $$m\frac{d^{2}r}{dt^{2}}=-\frac{\partial U}{\partial r}.$$

Force Field U can be represented as the sum of a bonded and a nonbonded contribution (Equation 26):

(26) $$U=U^{\mathrm{bonded}}+U^{\mathrm{nonbonded}}.$$

$U^{\mathrm{bonded}}$ groups potential energy involving directly bonded particles, such as bond stretching and angle bending (Equation 27):

(27) $$U^{\mathrm{bonded}}=\sum_{\mathrm{bonds}}\frac{k_{ij}^{b}}{2}{\left(d_{ij}-d_{ij}^{eq}\right)}^{2}+\sum_{\mathrm{angles}}\frac{k_{ij}^{\theta }}{2}{\left(cos\theta_{ijk}-cos\theta_{ijk}^{eq}\right)}^{2},$$

where $d_{ij}$ and $d_{ij}^{eq}$ are the bond distance and the bond distance at the equilibrium, respectively, between i and j particles, and $\theta_{ijk}$ and $\theta_{ijk}^{eq}$ are the angles and the angles at the equilibrium, respectively, between three particles i, j and k. Force constants for bonds and angles are represented by $k_{ij}^{b}$ and $k_{ij}^{\theta }$ respectively.

$U^{\mathrm{nonbonded}}$ force field includes all the other interactions between nondirectly bonded particles, expressed as the sum of Lennard–Jones 12–6 potentials and electrostatic interactions (Equation 28):

(28) $$U^{\mathrm{nonbonded}}=\sum_{i=1}^{n}\sum_{j\neq i}^{n}\left(4\varepsilon_{ij}\left[{\left(\frac{\sigma_{ij}}{r_{ij}}\right)}^{12}\;-{\left(\frac{\sigma_{ij}}{r_{ij}}\right)}^{6}\right]+\frac{q_{i}q_{j}}{4\pi \varepsilon_{0}r_{ij}}\right),$$

where $\sigma_{ij}$ and $\varepsilon_{ij}$ are the Lennard–Jones parameters and $q_{i}$ and $q_{j}$ are the partial charges of particles i and j (Brasiello et al. 2011; Cordina et al. 2023a).

This section of the review will focus on the most recent progress on CG‐MD and on the experimental data that were used to fit the developed models. For a more comprehensive description of the evolution of molecular dynamics from the atomistic to the CG model, the reader can refer to a recent review (Cordina et al. 2024).

### 4.2 Recent Progress in MD Model Development

One of the earliest MD simulations applied to a TAG system is reported by Chandrasekhar and Van Gunsteren (2002). In this paper, six different GROMOS parameter sets were compared. GROMOS is a forcefield originally developed for aliphatic alkanes. These parameter sets were applied to a trioctanoin molecule in the α phase, and the average cross‐sectional area per chain, which was used as an order indicator, was monitored, showing how slight variations in the forcefield could affect the outcome of the simulation.

MD simulations describing the effect of pressure and temperature on TAGs were performed by Greiner et al. (2012). In this work, several saturated pure TAGs and their mixtures were investigated. β crystals were built based on experimental crystal structures and then subjected to heating and to increasing pressure protocols. Densities and self‐diffusion coefficients were monitored and found to be in good agreement with experimental values. An attempt to simulate the solid–liquid interface of tripalmitin is reported: although it was not possible to simulate crystal growth, a dissolution of the crystal could be simulated.

In CG‐MD, the choice of a mapping scheme is of fundamental importance. Increasingly detailed mapping schemes for increasingly complex TAG systems have been reported over the years.

One of the first papers on the development of CG‐MD applied to TAGs was published by Brasiello et al. (2011). In this work, several increasingly detailed mapping schemes are proposed for tridecanoin. The scheme that distinguished between the ester group in position sn‐2 and the ones in positions n‐1 and sn‐3, while the methyl chains were mapped as two different beads: one for the three terminal carbons and another for the three central carbons, for a total of four beads, performed better. A simulation of the liquid–solid transition was carried out, and an α form formation was reported for the more complex mapping. The α polymorph was identified by the correct simulation of its temperature stability, packing, and chain distance and conformational angle values.

**FIGURE 5:**
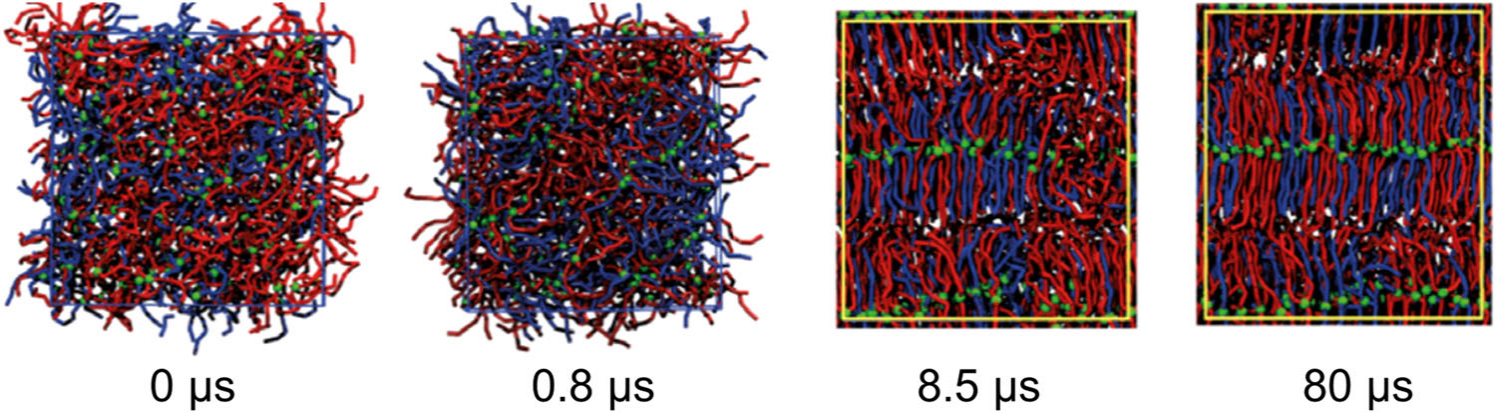
Evolution of the simulated molecular packing of a binary mixture of Tristearin (SSS) in red and tripalmitin (PPP) in blue with $X_{\mathrm{PPP}}=0.4$ during a cooling program. *Source*: Adapted from Pizzirusso et al. (2015).

This bead mapping was applied to a more complex system and reported in another paper (Pizzirusso et al. 2015). Larger molecules, such as tristearin and tripalmitin, both as pure compounds and as binary mixtures, were simulated. A fast‐cooling ramp and a step cooling profile, both with very high undercooling (around −100°C), were applied to the system to promote the formation of solid crystals. A graphic visualization of the binary mixture packing is reported in Figure 5. Such a high degree of undercooling was chosen to increase the probability of a rare nucleation event, but not enough to completely stop the TAGs’ mobility. To evaluate the packing order, both the radial distribution function (RDF) and the density isosurface plots were calculated. A density iso‐surface plot highlights the points inside the simulation box where a higher density, compared with the bulk average, is present, enabling the visualization of crystal plane formation. The reported crystals of the mixtures were in the α polymorph. The simulated crystal TAG conformations (e.g., tuning‐fork, chair, trident) over time were also monitored.

**FIGURE 6:**
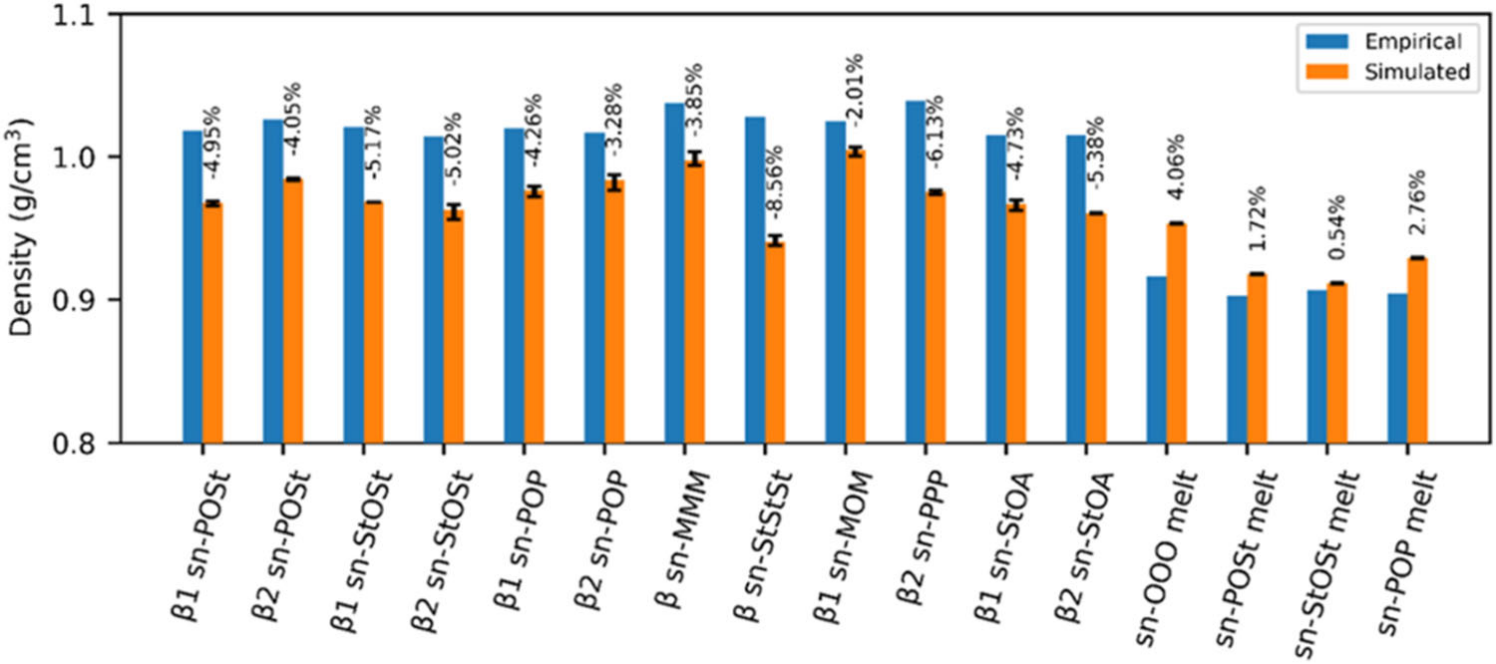
Empirical and coarse‐grained simulated densities for selected TAGs (crystal polymorphs and melts). Labels indicate % difference from empirical (Cordina et al. 2023a).

Further progress in the coarse‐graining mapping was reported by Cordina et al. (2023a). They developed a new CG forcefield, which was also able to simulate monounsaturated TAGs thanks to a more elaborate bead mapping. The new nonbonded parameters needed for this new mapping scheme were parametrized through experimental data of lattice type and densities of both crystalline and molten TAGs. This bead mapping scheme was used to investigate the melting behavior of pure unsaturated TAGs and their mixtures. Empirical and simulated densities of various monounsaturated TAG crystals and melts are reported in Figure 6.

In later papers from the same group, this forcefield was used to simulate several unsaturated TAG systems. In Cordina and coworkers’ research (Cordina et al. 2023c), the simulations of TAG crystals melting are reported. Several unsaturated pure TAG system crystals are built by repeating single coarse molecules. To better predict the real crystallization behavior, voids were generated in the crystal lattice during the simulation of a heating profile.

The previous approach for simulating TAG crystals and their melting behavior was used in a later work from the same group (Cordina et al. 2023b). In this work, the nonlinear melting behavior of an unsaturated binary TAG mixture is reported. Specifically, the melting simulations of POP/POS, POP/SOS, and POS/SOS blends, related to cocoa butter composition, were investigated. The experimental melting trend was replicated correctly, although the simulated temperatures were generally slightly lower than the experimental values. The authors explained this behavior due to the presence of mini‐voids created in the crystal lattice by the difference in length between the FA chains of the two TAGs.

### 4.3 Molecular Models Validation

To validate the bead mapping scheme reported by Brasiello et al. (2011), literature experimental data were used. Viscosity data were obtained through kinematic viscosimetry (Valeri and Meirelles 1997), while density data were collected with a pycnometer (Noureddini et al. 1992). The authors report that simulated values were in good agreement with the density data, while shear viscosity predictions were found to be less accurate. The same mapping scheme was used to perform a CG‐MD simulation of the crystallization of tridecanoin. The interchain distance during crystallization was computed and compared with values obtained through X‐ray diffraction, which confirmed the formation of an α polymorph.

Experimental and simulated α polymorph melting was compared by Pizzirusso et al. (2015). This estimation was performed for both pure and mixed systems of SSS and PPP. The literature DSC data from (Costa et al. 2010) were used for comparison. While the general trend was replicated successfully for different fractions, a systematic difference of 89 K between the experimental and simulated melting point values cannot be ignored. This difference is ascribed by the authors to the intrinsic limitation of CG models to predict exact values. Later work from the same group (Pizzirusso et al. 2018) presented a simulation of a β SSS—β PPP system. WAXS was used to confirm the β polymorphism, and SAXS was used to confirm the long d‐spacing of the simulated system, while melting points were detected with DSC. The simulated eutectic concentration matched the experimental one, but the modeled temperature showed an offset of about 35°C.

Cordina et al. (2023a) validated their newly developed force field using literature density values obtained with a gas pycnometer (Arishima et al. 1995), which were found to be in good agreement (less than 6% difference) with the simulated data. Also, literature‐available cell dimensions (Van Mechelen et al. 2006) and angles for the β polymorph were used for the model validation. This model was able to estimate the density and cell dimensions of different unsaturated TAGs from those used for the parametrization.

The random distribution of SSS and PPP in the mixture reported by Cordina et al. (2023b) was confirmed by comparing simulated and experimental wide‐angle X‐ray scattering diffractograms. The melting process was monitored by simulated and experimental DSC and crystal density data.

### 4.4 Current Knowledge Gaps and Future Trends for Molecular Modeling of Fats

Coarse‐grained molecular dynamics is one of the most promising techniques to model fat crystallization at the molecular scale. Different from thermodynamic and kinetic modeling, MD is the only technique that can predict structural characteristics of TAG crystals from a molecular point of view. Knowledge of structure, and consequently polymorphism and miscibility, could be of great value in food, cosmetics, and pharmaceutical product design.

A significant amount of attention has been dedicated to developing reasonable mapping schemes to describe saturated TAGs, and only in recent years has the methodology been improved to model unsaturated TAGs.

Most of the crystallization simulations reported refer to the formation of α polymorphs, which are less stable and thus less practically useful. The β polymorph was not directly obtained by simulating the crystallization process; instead, its melting process was simulated after building its structure based on known crystallographic data. CG‐MD simulations of the melting process of the aforementioned crystals were conducted. Experimental melting trends were replicated, but the experimental temperatures were often underestimated. To the best of our knowledge, molecular simulations of TAGs in the β′ polymorph have not been reported in the literature, despite the importance of this particular polymorph in the food industry.

The cited works about TAG modeling are based on the simulation of pure TAGs or their binary mixtures. This is a very important starting point, but a simulation of a real fat mixture, due to its complex compositional profile, is still far from being achieved. Despite these challenges, even simulations of pure TAG or simple mixtures are useful in the sense that they could be an alternative to experiments and classic thermodynamic models to obtain thermodynamic parameters to be used as inputs in the thermodynamic models reported in Section 2.

## 5 Conclusion

Fat crystallization modeling is of utmost importance to better design efficient and sustainable food manufacturing processes. This review reported the progress on crystallization modeling from a thermodynamic, kinetic, and molecular point of view applied to different fat systems.

Despite the recent advancements in each type of modeling technique, it is clear that a single modeling approach alone is not able to confidently reproduce all the features of the crystallization behavior of complex TAG mixtures. Nevertheless, each modeling strategy can be useful in describing part of the fat crystallization behavior.

Thermodynamic modeling is useful to describe equilibrium phase diagrams, which are important for product functionality. In fact, they can be used to quantitatively calculate the SFC curve of a fat mixture provided that the thermal properties of pure TAGs and binary interaction parameters are available. Kinetic models are fundamental to quantifying crystallization phenomena and designing efficient manufacturing processes. Crystallization curves for thermodynamic and kinetic models can be obtained by many different experimental techniques, from simple bench instruments to synchrotron‐based experiments. It should be noted that at the moment, X‐ray scattering is the only technique able to unequivocally identify polymorphs, and the precious information that this technique gives should be integrated into models for a comprehensive description of kinetics.

Molecular models have the potential to simulate the crystallization phenomena at the molecular level, perhaps providing data for the thermodynamic models (e.g., melting temperatures and enthalpies).

Synergy between different models and reliable experimental data is of fundamental importance for the future of modeling of fat crystallization. It must be noted that, at the moment, a comprehensive modeling workflow for fat mixtures is still far from being achieved due to their compositional complexity. Indeed, natural fats often present hundreds of different TAGs and are rich in unsaturated FA; for this reason, mixtures are often simplified in the models, not including the less abundant TAGs, which can impact the real crystallization behavior.

Additionally, many of the presented models are empirical and rely on pure component data; this is often incomplete in the literature (e.g., lack of information for all the possible polymorphs, lack of information on unsaturated TAGs). Moreover, very few data are available to describe mixing behavior and nonideality of binary TAG mixtures for the estimation of activity coefficients.

Modeling applied to TAGs and their mixtures is a subject of great interest due to its immediate application in food, cosmetic, and pharmaceutical applications. With the increasing growth of computational power and the increasing interest in fat‐based commodities (often of nonconventional origin), the interest in this field is expected to grow.

## Author Contributions

**Michele Lessona**: writing – original draft, investigation, conceptualization, data curation, methodology. **Antoine Cros**: funding acquisition, writing – review and editing, supervision, methodology. **Laurent Sagalowicz**: funding acquisition, supervision, methodology, writing – review and editing. **Yogesh Harshe**: methodology, writing – review and editing, supervision. **Antonio Buffo**: supervision, resources, writing – review and editing, investigation, conceptualization, methodology, validation. **Elena Simone**: conceptualization, investigation, writing – review and editing, supervision, resources, methodology, validation.

## Conflicts of Interest

The authors declare no conflicts of interest.

## References

[crf370315-bib-0001] Acevedo, N. C. , and A. G. Marangoni . 2015. “Nanostructured Fat Crystal Systems.” Annual Review of Food Science and Technology 6, no. 1: 71–96. 10.1146/annurev-food-030713-092400. 25422879

[crf370315-bib-0002] Andrade, S. S. , R. S. B. Ferreira , F. O. Farias , et al. 2023. “Solid‐Liquid Equilibria of Triacylglycerols and Vitamin E Mixtures.” Food Research International 173: 113440. 10.1016/j.foodres.2023.113440. 37803766

[crf370315-bib-0003] Arishima, T. , N. Sagi , H. Mori , and K. Sato . 1995. “Density Measurement of the Polymorphic Forms of POP, POS and SOS.” Journal of Japan Oil Chemists' Society 44, no. 6: 431–437. 10.5650/jos1956.44.431.

[crf370315-bib-0004] Arita‐Merino, N. , H. van Valenberg , E. P. Gilbert , and E. Scholten . 2020. “Quantitative Phase Analysis of Complex Fats During Crystallization.” Crystal Growth & Design 20, no. 8: 5193–5202. 10.1021/acs.cgd.0c00416.

[crf370315-bib-0005] Avrami, M. 1939. “Kinetics of Phase Change. I: General Theory.” The Journal of Chemical Physics 7, no. 12: 1103–1112. 10.1063/1.1750380.

[crf370315-bib-0006] Avrami, M. 1940. “Kinetics of Phase Change. II Transformation‐Time Relations for Random Distribution of Nuclei.” The Journal of Chemical Physics 8, no. 2: 212–224. 10.1063/1.1750631.

[crf370315-bib-0007] Bayés‐García, L. , T. Calvet , M. À. Cuevas‐Diarte , S. Ueno , and K. Sato . 2013. “Crystallization and Transformation of Polymorphic Forms of Trioleoyl Glycerol and 1,2‐Dioleoyl‐3‐rac‐linoleoyl Glycerol.” The Journal of Physical Chemistry B 117, no. 31: 9170–9181. 10.1021/jp403872a. 23841675

[crf370315-bib-0008] Bayés‐García, L. , K. Sato , and S. Ueno . 2020. “Polymorphism of Triacylglycerols and Natural Fats.” In Bailey's Industrial Oil and Fat Products, (1–49). Wiley. 10.1002/047167849X.bio020.pub2.

[crf370315-bib-0009] Berg, T. G. O. , and U. I. Brimberg . 1983. “Über die Kinetik der Fettkristallisation.” Fette, Seifen, Anstrichmittel 85, no. 4: 142–149. 10.1002/lipi.19830850402.

[crf370315-bib-0010] Bölük, E. , E. Akdeniz , R. Gunes , I. Palabiyik , N. Konar , and O. S. Toker . 2024. “Determination of the Process Effect on Cocoa Butter Crystallization by Rheometer: Kinetic Modeling by Gompertz Equation.” Journal of Food Science 89, no. 5: 2867–2878. 10.1111/1750-3841.17040. 38551060 PMC13281141

[crf370315-bib-0011] Bootello, M. A. , R. W. Hartel , M. Levin , et al. 2013. “Studies of Isothermal Crystallisation Kinetics of Sunflower Hard Stearin‐Based Confectionery Fats.” Food Chemistry 139, no. 1–4: 184–195. 10.1016/j.foodchem.2012.11.141. 23561095

[crf370315-bib-0012] Brasiello, A. , S. Crescitelli , and G. Milano . 2011. “Development of a Coarse‐grained Model for Simulations of Tridecanoin Liquid–Solid Phase Transitions.” Physical Chemistry Chemical Physics 13, no. 37: 16618. 10.1039/c1cp20604d. 21858376

[crf370315-bib-0013] Brasiello, A. , S. Crescitelli , and G. Milano . 2012. “A Multiscale Approach to Triglycerides Simulations: From Atomistic to Coarse‐Grained Models and Back.” Faraday Discussions 158: 479. 10.1039/c2fd20037f. 23234181

[crf370315-bib-0014] *Breaking Down Fats and Oils* . (Issue July). 2021. https://www.forumforthefuture.org/breaking-down-fats-and-oils-report.

[crf370315-bib-0015] Brinkmann, J. , C. Luebbert , D. H. Zaitsau , S. P. Verevkin , and G. Sadowski . 2019. “Thermodynamic Modeling of Triglycerides Using PC‐SAFT.” Journal of Chemical & Engineering Data 64, no. 4: 1446–1453. 10.1021/acs.jced.8b01046.

[crf370315-bib-0016] Burton, W. K. , N. Cabrera , and F. C. Frank . 1951. “The Growth of Crystals and the Equilibrium Structure of Their Surfaces.” Philosophical Transactions of the Royal Society of London Series A, Mathematical and Physical Sciences 243, no. 866: 299–358. 10.1098/rsta.1951.0006. PMC4360084 25750141

[crf370315-bib-0017] Chandrasekhar, I. , and W. F. Van Gunsteren . 2002. “A Comparison of the Potential Energy Parameters of Aliphatic Alkanes: Molecular Dynamics Simulations of Triacylglycerols in the Alpha Phase.” European Biophysics Journal 31, no. 2: 89–101. 10.1007/s00249-001-0196-9. 12012112

[crf370315-bib-0018] Cordina, R. J. , B. Smith , and T. Tuttle . 2023a. “COGITO: A Coarse‐Grained Force Field for the Simulation of Macroscopic Properties of Triacylglycerides.” Journal of Chemical Theory and Computation 19, no. 4: 1333–1341. 10.1021/acs.jctc.2c00975. 36728833 PMC9979597

[crf370315-bib-0019] Cordina, R. J. , B. Smith , and T. Tuttle . 2023b. “Predicting Lipid Eutectics Using Coarse‐Grained Molecular Dynamics.” The Journal of Physical Chemistry B 127, no. 47: 10236–10242. 10.1021/acs.jpcb.3c06297. 37975801 PMC10694813

[crf370315-bib-0020] Cordina, R. J. , B. Smith , and T. Tuttle . 2023c. “Triacylglyceride Melting Point Determination Using Coarse‐Grained Molecular Dynamics.” Journal of Computational Chemistry 44, no. 21: 1795–1801. 10.1002/jcc.27128. 37163230

[crf370315-bib-0021] Cordina, R. J. , B. Smith , and T. Tuttle . 2024. “Mathematical and Computational Modeling of Fats and Triacylglycerides.” Comprehensive Reviews in Food Science and Food Safety 23, no. 2: e13316. 10.1111/1541-4337.13316. 38506169

[crf370315-bib-0022] Costa, M. C. , L. A. D. Boros , M. P. Rolemberg , M. A. Krähenbühl , and A. J. A. Meirelles . 2010. “Solid‐Liquid Equilibrium of Saturated Fatty Acids + Triacylglycerols.” Journal of Chemical and Engineering Data 55, no. 2: 974–977. 10.1021/je900410j.

[crf370315-bib-0023] Costa, M. C. , M. Sardo , M. P. Rolemberg , et al. 2009. “The Solid‐Liquid Phase Diagrams of Binary Mixtures of Consecutive, Even Saturated Fatty Acids: Differing by Four Carbon Atoms.” Chemistry and Physics of Lipids 157, no. 1: 40–50. 10.1016/j.chemphyslip.2008.09.006. 18996101

[crf370315-bib-0024] Coutinho, J. A. P. 1998. “Predictive UNIQUAC: A New Model for the Description of Multiphase Solid−Liquid Equilibria in Complex Hydrocarbon Mixtures.” Industrial & Engineering Chemistry Research 37, no. 12: 4870–4875. 10.1021/ie980340h.

[crf370315-bib-0025] Dewettinck, K. , I. Foubert , M. Basiura , and B. Goderis . 2004. “Phase Behavior of Cocoa Butter in a Two‐step Isothermal Crystallization.” Crystal Growth and Design 4, no. 6: 1295–1302. 10.1021/cg049772n.

[crf370315-bib-0026] Dibildox‐Alvarado, E. , and J. F. Toro‐Vazquez . 1998. “Evaluation of Tripalmitin Crystallization in Sesame Oil Through a Modified Avrami Equation.” Journal of the American Oil Chemists' Society 75, no. 1: 73–76. 10.1007/s11746-998-0013-z.

[crf370315-bib-0027] Euston, S. R. 2013. “Modelling and Computer Simulation of Food Structures.” In Food Microstructures, 336–385. Elsevier. 10.1533/9780857098894.2.336.

[crf370315-bib-0028] Foster, R. , C. S. Williamson , and J. Lunn . 2009. “Culinary Oils and Their Health Effects.” Nutrition Bulletin 34, no. 1: 4–47. 10.1111/j.1467-3010.2008.01738.x.

[crf370315-bib-0029] Foubert, I. , K. Dewettinck , G. Janssen , and P. A. Vanrolleghem . 2006. “Modelling Two‐Step Isothermal Fat Crystallization.” Journal of Food Engineering 75, no. 4: 551–559. 10.1016/j.jfoodeng.2005.04.038.

[crf370315-bib-0030] Foubert, I. , K. Dewettinck , and P. A. Vanrolleghem . 2003. “Modelling of the Crystallization Kinetics of Fats.” Trends in Food Science & Technology 14, no. 3: 79–92. 10.1016/S0924-2244(02)00256-X.

[crf370315-bib-0031] Foubert, I. , P. A. Vanrolleghem , B. Vanhoutte , and K. Dewettinck . 2002. “Dynamic Mathematical Model of the Crystallization Kinetics of Fats.” Food Research International 35, no. 10: 945–956. 10.1016/S0963-9969(02)00157-6.

[crf370315-bib-0032] Garti, N. , and K. Satō . 2001. Crystallization Processes in Fats and Lipid Systems, edited by N. Garti and K. Sato , CRC Press. 10.1201/9781482270884.

[crf370315-bib-0033] Geary, M. , and R. Hartel . 2017. “Crystallization Behavior and Kinetics of Chocolate‐Lauric Fat Blends and Model Systems. *JAOCS* .” Journal of the American Oil Chemists' Society 94, no. 5: 683–692. 10.1007/s11746-017-2973-3.

[crf370315-bib-0034] Ghazani, S. M. , and A. G. Marangoni . 2021. “Molecular Origins of Polymorphism in Cocoa Butter.” Annual Review of Food Science and Technology 12: 567–590. 10.1146/annurev-food-070620-022551. 33467907

[crf370315-bib-0035] Golodnizky, D. , and M. Davidovich‐Pinhas . 2022. “New Insights Into the Thermodynamics and Kinetics of Triacylglycerols Crystallization.” Innovative Food Science & Emerging Technologies 81: 103115. 10.1016/j.ifset.2022.103115.

[crf370315-bib-0036] Gompertz, B. 1825. “XXIV. On the Nature of the Function Expressive of the Law of Human Mortality, and on a New Mode of Determining the Value of Life Contingencies. In a Letter to Francis Baily, Esq. F. R. S. &c.” Philosophical Transactions of the Royal Society of London 115: 513–583. 10.1098/rstl.1825.0026. PMC4360127 25750242

[crf370315-bib-0037] Goto, M. , D. R. Kodali , D. M. Small , K. Honda , K. Kozawa , and T. Uchida . 1992. “Single Crystal Structure of a Mixed‐Chain Triacylglycerol: 1,2‐Dipalmitoyl‐3‐Acetyl‐sn‐Glycerol.” Proceedings of the National Academy of Sciences 89, no. 17: 8083–8086. 10.1073/pnas.89.17.8083. PMC49860 1518834

[crf370315-bib-0038] Greiner, M. , A. M. Reilly , and H. Briesen . 2012. “Temperature‐ and Pressure‐Dependent Densities, Self‐Diffusion Coefficients, and Phase Behavior of Monoacid Saturated Triacylglycerides: Toward Molecular‐Level Insights Into Processing.” Journal of Agricultural and Food Chemistry 60, no. 20: 5243–5249. 10.1021/jf3004898. 22500590

[crf370315-bib-0039] Hernqvist, L. , and K. Larsson . 1982. “On the Crystal Structure of the β′‐Form of Triglycerides and Structural Changes at the Phase Transitions LIQ. → α → β′ ← β.” Fette, Seifen, Anstrichmittel 84, no. 9: 349–354. 10.1002/lipi.19820840905.

[crf370315-bib-0040] Himawan, C. , V. M. Starov , and A. G. F. Stapley . 2006. “Thermodynamic and Kinetic Aspects of Fat Crystallization.” Advances in Colloid and Interface Science 122, no. 1–3: 3–33. 10.1016/j.cis.2006.06.016. 16904622

[crf370315-bib-0041] Hjorth, J. L. , R. L. Miller , J. M. Woodley , and S. Kiil . 2015a. “Kinetic Modeling of Multi‐Component Crystallization of Industrial‐Grade Oils and Fats.” European Journal of Lipid Science and Technology 117, no. 7: 1066–1078. 10.1002/ejlt.201400263.

[crf370315-bib-0042] Hjorth, J. L. , R. L. Miller , J. M. Woodley , and S. Kiil . 2015b. “Thermodynamic Modeling of Multi‐Phase Solid–Liquid Equilibria in Industrial‐Grade Oils and Fats.” Journal of the American Oil Chemists' Society 92, no. 1: 17–28. 10.1007/s11746-014-2577-0.

[crf370315-bib-0043] Idziak, S. H. J. 2018. “Powder X‐Ray Diffraction of Triglycerides in the Study of Polymorphism.” In Structure‐Function Analysis of Edible Fats, 73–99. Elsevier. 10.1016/B978-0-12-814041-3.00003-4.

[crf370315-bib-0044] Ioannidi, E. , E. Aarøe , A. de Juan , J. Risbo , and F. W. J. van den Berg . 2023. “Modeling Changes in Chocolate During Production and Storage by ATR‐FT‐IR Spectroscopy and MCR‐ALS Hybrid Soft and Hard Modeling.” Chemometrics and Intelligent Laboratory Systems 233, no. December 2022: 104735. 10.1016/j.chemolab.2022.104735.

[crf370315-bib-0045] Jensen, L. H. , and A. J. Mabis . 1966. “Refinement of the Structure of β‐Tricaprin.” Acta Crystallographica 21, no. 5: 770–781. 10.1107/S0365110x66003839. 6013555

[crf370315-bib-0046] Khanna, Y. P. , and T. J. Taylor . 1988. “Comments and Recommendations on the Use of the Avrami Equation for Physico‐Chemical Kinetics.” Polymer Engineering & Science 28, no. 16: 1042–1045. 10.1002/pen.760281605.

[crf370315-bib-0047] Klamt, A. 1995. “Conductor‐Like Screening Model for Real Solvents: A New Approach to the Quantitative Calculation of Solvation Phenomena.” The Journal of Physical Chemistry 99, no. 7: 2224–2235. 10.1021/j100007a062.

[crf370315-bib-0048] Klamt, A. , V. Jonas , T. Bürger , and J. C. W. Lohrenz . 1998. “Refinement and Parametrization of COSMO‐RS.” The Journal of Physical Chemistry A 102, no. 26: 5074–5085. 10.1021/jp980017s.

[crf370315-bib-0049] Kloek, W. , P. Walstra , and T. van Vliet . 2000. “Crystallization Kinetics of Fully Hydrogenated Palm Oil in Sunflower Oil Mixtures.” Journal of the American Oil Chemists' Society 77, no. 4: 389–398. 10.1007/s11746-000-0063-z.

[crf370315-bib-0050] Ladd Parada, M. , M. J. Povey , J. Vieira , M. Rappolt , and M. E. Ries . 2019. “Early Stages of Fat Crystallisation Evaluated by Low‐field NMR and Small‐angle X‐ray Scattering.” Magnetic Resonance in Chemistry 57, no. 9: 686–694. 10.1002/mrc.4860. 30843260

[crf370315-bib-0051] Larsen, B. L. , P. Rasmussen , and A. Fredenslund . 1987. “A Modified UNIFAC Group‐contribution Model for Prediction of Phase Equilibria and Heats of Mixing.” Industrial & Engineering Chemistry Research 26, no. 11: 2274–2286. 10.1021/ie00071a018.

[crf370315-bib-0052] Larsson, K. , S. J. Cyvin , L. Rymo , et al. 1966. “Classification of Glyceride Crystal Forms.” Acta Chemica Scandinavica 20: 2255–2260. 10.3891/acta.chem.scand.20-2255. 5966871

[crf370315-bib-0053] Lewis, M. M. 2014. “Understanding Nutrition.” Journal of Nutrition Education and Behavior 46, no. 1: 88.e7. 10.1016/j.jneb.2013.07.005.

[crf370315-bib-0054] Lin, S.‐T. , and S. I. Sandler . 2002. “A Priori Phase Equilibrium Prediction From a Segment Contribution Solvation Model.” Industrial & Engineering Chemistry Research 41, no. 5: 899–913. 10.1021/ie001047w.

[crf370315-bib-0055] Los, J. , and E. Flöter . 1999. “Construction of Kinetic Phase Diagrams.” Physical Chemistry Chemical Physics 1, no. 18: 4251–4257. 10.1039/a903245b.

[crf370315-bib-0056] Macias‐Rodriguez, B. A. , and A. A. Marangoni . 2018. “Linear and Nonlinear Rheological Behavior of Fat Crystal Networks.” Critical Reviews in Food Science and Nutrition 58, no. 14: 2398–2415. 10.1080/10408398.2017.1325835. 28471278

[crf370315-bib-0057] Marangoni, A. G. 1998. “On the Use and Misuse of the Avrami Equation in Characterization of the Kinetics of Fat Crystallization.” In Press 1465 JAOCS, (Vol. 75, Issue no. 10:).

[crf370315-bib-0058] Marangoni, A. G. 2018. Structure‐Function Analysis of Edible Fats. Elsevier. 10.1016/C2017-0-00579-7.

[crf370315-bib-0059] Marangoni, A. G. , S. M. Ghazani , and E. Pensini . 2023. “An Entropy‐Centric Equilibrium Cooperative Theory for the Melting Behavior of Nonideal Triaclylglycerol Mixtures.” Journal of the American Oil Chemists' Society 100, no. 2: 107–122. 10.1002/aocs.12669.

[crf370315-bib-0060] Marangoni, A. G. , and D. Rousseau . 1996. “Is Plastic Fat Rheology Governed by the Fractal Nature of the Fat Crystal Network?.” Journal of the American Oil Chemists' Society 73, no. 8: 991–994.

[crf370315-bib-0061] Marangoni, A. G. , and L. H. Wesdorp . 2012. “Structure and Properties of Fat Crystal Networks.” In Modifying Lipids for Use in Food. CRC Press. 10.1201/b12883.

[crf370315-bib-0062] Marty‐Terrade, S. , and A. G. Marangoni . 2012. “Impact of Cocoa Butter Origin on Crystal Behavior.” Cocoa Butter and Related Compounds 245–274. 10.1016/B978-0-9830791-2-5.50014-1.

[crf370315-bib-0063] Maximo, G. J. , M. C. Costa , J. A. P. Coutinho , and A. J. A. Meirelles . 2014. “Trends and Demands in the Solid–Liquid Equilibrium of Lipidic Mixtures.” RSC Advances 4, no. 60: 31840–31850. 10.1039/C4RA02715A.

[crf370315-bib-0064] Mazzanti, G. , A. G. Marangoni , and S. H. J. Idziak . 2009. “Synchrotron Study on Crystallization Kinetics of Milk Fat Under Shear Flow.” Food Research International 42, no. 5–6: 682–694. 10.1016/j.foodres.2009.02.009.

[crf370315-bib-0065] Metilli, L. , L. Morris , A. Lazidis , et al. 2022. “Real‐time Monitoring of Fat Crystallization Using Pulsed Acoustic Spectroscopy and Supervised Machine Learning.” Journal of Food Engineering 335: 111192. 10.1016/j.jfoodeng.2022.111192.

[crf370315-bib-0066] Minato, A. , S. Ueno , K. Smith , Y. Amemiya , and K. Sato . 1997. “Thermodynamic and Kinetic Study on Phase Behavior of Binary Mixtures of POP and PPO Forming Molecular Compound Systems.” The Journal of Physical Chemistry B 101, no. 18: 3498–3505. 10.1021/jp962956v.

[crf370315-bib-0067] Miyagawa, Y. 2021. “Crystallization Kinetics of Low‐Melting Point Vegetable Oils.” Japan Journal of Food Engineering 22, no. 3: 21596. 10.11301/jsfe.21596.

[crf370315-bib-0068] Miyagawa, Y. , M. Yoshida , K. Nakagawa , and S. Adachi . 2018. “Kinetic Analysis of Rapeseed Oil Crystallization During Isothermal Storage.” Crystal Growth & Design 18, no. 2: 642–650. 10.1021/acs.cgd.7b00789.

[crf370315-bib-0069] Moorthy, A. S. , R. Liu , G. Mazzanti , L. H. Wesdorp , and A. G. Marangoni . 2017. “Estimating Thermodynamic Properties of Pure Triglyceride Systems Using the Triglyceride Property Calculator.” Journal of the American Oil Chemists' Society . 10.1007/s11746-016-2935-1.

[crf370315-bib-0070] Nakanishi, K. , Y. Mikiya , T. Ishiguro , and S. Ueno . 2018. “Crystallization Behavior of Molecular Compound in Binary Mixture System of 1,3‐Dioleoyl‐2‐Palmitoyl‐sn ‐Glycerol and 1,3‐Dipalmitoyl‐2‐Oleoyl‐ sn‐Glycerol.” Journal of the American Oil Chemists' Society 95, no. 1: 51–59. 10.1002/aocs.12005.

[crf370315-bib-0071] Narine, S. S. , and A. G. Marangoni . 1999. “Relating Structure of Fat Crystal Networks to Mechanical Properties.” Food Research International 32, no. 4: 227–248. 10.1016/S0963-9969(99)00078-2.

[crf370315-bib-0072] Noureddini, H. , B. C. Teoh , and D. Clements . 1992. Densities of Vegetable Oils and Fatty Acids . https://digitalcommons.unl.edu/chemeng_biomaterials.

[crf370315-bib-0073] Ollivon, M. , G. Keller , C. Bourgaux , D. Kalnin , P. Villeneuve , and P. Lesieur . 2006. “DSC and High Resolution X‐Ray Diffraction Coupling.” Journal of Thermal Analysis and Calorimetry 85, no. 1: 219–224. 10.1007/s10973-005-7351-y.

[crf370315-bib-0074] Ollivon, M. , and R. Perron . 1982. “Measurements of Enthalpies and Entropies of Unstable Crystalline Forms of Saturated Even Monoacid Triglycerides.” Thermochimica Acta 53, no. 2: 183–194. 10.1016/0040-6031(82)85007-7.

[crf370315-bib-0075] Parcell, J. , Y. Kojima , A. Roach , and W. Cain . 2018. “Global Edible Vegetable Oil Market Trends.” Biomedical Journal of Scientific & Technical Research 2, no. 1: 00. 10.26717/BJSTR.2018.02.000680.

[crf370315-bib-0076] Pereira, E. , F. T. Junqueira , A. J. Meirelles , A. de , and G. J. Maximo . 2019. “Prediction of the Melting Behavior of Edible Fats Using UNIFAC and UNIQUAC Models.” Fluid Phase Equilibria 493: 58–66. 10.1016/j.fluid.2019.04.004.

[crf370315-bib-0077] Pereira, E. , D. T. V. Pereira , A. J. A. Meirelles , and G. J. Maximo . 2022. “Modeling the Solid‐Liquid Equilibrium of Binary Mixtures of Triacylglycerols Using UNIFAC and Predictive UNIQUAC Models.” Fluid Phase Equilibria 554: 113327. 10.1016/j.fluid.2021.113327.

[crf370315-bib-0078] Pizzirusso, A. , A. Brasiello , A. D. Nicola , A. G. Marangoni , and G. Milano . 2015. “Coarse‐grained Modelling of Triglyceride Crystallisation: a Molecular Insight Into Tripalmitin Tristearin Binary Mixtures by Molecular Dynamics Simulations.” Journal of Physics D: Applied Physics 48, no. 49: 494004. 10.1088/0022-3727/48/49/494004.

[crf370315-bib-0079] Pizzirusso, A. , F. Peyronel , E. D. Co , A. G. Marangoni , and G. Milano . 2018. “Molecular Insights Into the Eutectic Tripalmitin/Tristearin Binary System.” Journal of the American Chemical Society 140, no. 39: 12405–12414. 10.1021/jacs.8b04729. 30178998

[crf370315-bib-0080] Povey, M. J. W. , T. S. Awad , R. Huo , and Y. Ding . 2009. “Quasi‐isothermal Crystallisation Kinetics, Non‐Classical Nucleation and Surfactant‐Dependent Crystallisation of Emulsions.” European Journal of Lipid Science and Technology 111, no. 3: 236–242. 10.1002/ejlt.200800193.

[crf370315-bib-0081] Pratama, Y. , E. Simone , and M. Rappolt . 2021. “The Unique Crystallization Behavior of Buffalo Milk Fat.” Crystal Growth and Design 21, no. 4: 2113–2127. 10.1021/acs.cgd.0c01543.

[crf370315-bib-0082] Rebry, F. , A. Declerck , K.‐F. Ratzsch , M. Wilhelm , K. Dewettinck , and P. Van der Meeren . 2021. “Rheo‐NMR to Investigate Fat Crystallization Under Shear.” Current Research in Food Science 4: 414–420. 10.1016/j.crfs.2021.05.004. 34195622 PMC8233192

[crf370315-bib-0083] Sasaki, M. , S. Ueno , and K. Sato . 2012. “Polymorphism and Mixing Phase Behavior of Major Triacylglycerols of Cocoa Butter.” Cocoa Butter and Related Compounds 151–172. 10.1016/B978-0-9830791-2-5.50009-8.

[crf370315-bib-0084] Sato, K. 2001. “Crystallization Behaviour of Fats and Lipids — A Review.” Chemical Engineering Science 56, no. 7: 2255–2265. 10.1016/S0009-2509(00)00458-9.

[crf370315-bib-0085] Sato, K. , and T. Kuroda . 1987. “Kinetics of Melt Crystallization and Transformation of Tripalmitin Polymorphs.” Journal of the American Oil Chemists' Society 64, no. 1: 124–127. 10.1007/BF02546266.

[crf370315-bib-0086] Schaink, H. M. 2023. “Calculation of the Solid Fat Content of Vegetable Fats Using the Hildebrand Equation.” Journal of the American Oil Chemists' Society 100, no. 12: 929–944. 10.1002/aocs.12724.

[crf370315-bib-0087] Seilert, J. , and E. Flöter . 2021. “A Configurational Approach to Model Triglyceride Pure Component Properties.” European Journal of Lipid Science and Technology 123, no. 10: 00. 10.1002/ejlt.202100010.

[crf370315-bib-0088] Seilert, J. , A. S. Moorthy , A. J. Kearsley , and E. Flöter . 2021. “Revisiting a Model to Predict Pure Triglyceride Thermodynamic Properties: Parameter Optimization and Performance.” Journal of the American Oil Chemists' Society 98, no. 8: 837–850. 10.1002/aocs.12515.

[crf370315-bib-0089] Seilert, J. , M. Rappolt , G. Dol , and E. Flöter . 2024. “Interplay of Polymorphic Transition and Mixed Crystal Formation in Model Fat Systems.” Crystal Growth and Design 24, no. 3: 1146–1158. 10.1021/acs.cgd.3c01164.

[crf370315-bib-0090] Simone, E. , M. Rappolt , H. Ewens , et al. 2024. “A Synchrotron X‐Ray Scattering Study of the Crystallization Behavior of Mixtures of Confectionary Triacylglycerides: Effect of Chemical Composition and Shear on Polymorphism and Kinetics.” Food Research International 177: 113864. 10.1016/j.foodres.2023.113864. 38225135

[crf370315-bib-0091] Szydłowska‐Czerniak, A. , D. Rabiej , and R. Pawłowicz . 2017. “Comparison of the Crystallization Behaviors of Different Types of Cocoa Butters and Chocolates.” Journal of Food Processing and Preservation 41, no. 5: 00. 10.1111/jfpp.13154.

[crf370315-bib-0092] Takeuchi, M. , S. Ueno , E. Flöter , and K. Sato . 2002. “Binary Phase Behavior of 1,3‐Distearoyl‐2‐oleoyl‐sn‐Glycerol (SOS) and 1,3‐Distearoyl‐2‐linoleoyl‐sn‐Glycerol (SLS).” In 627 JAOCS, (Vol. 79, Issue no. 7).

[crf370315-bib-0093] Takeuchi, M. , S. Ueno , and K. Sato . 2003. “Synchrotron Radiation SAXS/WAXS Study of Polymorph‐Dependent Phase Behavior of Binary Mixtures of Saturated Monoacid Triacylglycerols.” Crystal Growth and Design 3, no. 3: 369–374. 10.1021/cg025594r.

[crf370315-bib-0094] Teles dos Santos, M. , V. Gerbaud , and G. A. C. Le Roux . 2014. “Solid Fat Content of Vegetable Oils and Simulation of Interesterification Reaction: Predictions From Thermodynamic Approach.” Journal of Food Engineering 126: 198–205. 10.1016/j.jfoodeng.2013.11.012.

[crf370315-bib-0095] Teles dos Santos, M. , V. Gerbaud , and G. A. C. L. E. Roux . 2013. “Modeling and Simulation of Melting Curves and Chemical Interesterification of Binary Blends of Vegetable Oils.” Chemical Engineering Science 87: 14–22. 10.1016/j.ces.2012.09.026.

[crf370315-bib-0096] Teles dos Santos, M. , P. Morgavi , and G. A. C. Le Roux . 2018. “Exploring Amazonian Fats and Oils Blends by Computational Predictions of Solid Fat Content.” OCL 25, no. 1: D107. 10.1051/ocl/2017055.

[crf370315-bib-0097] Teles dos Santos, M. , I. S. Viana , J. N. R. Ract , and G. A. C. Le Roux . 2016. “Thermal Properties of Palm Stearin, Canola Oil and Fully Hydrogenated Soybean Oil Blends: Coupling Experiments and Modeling.” Journal of Food Engineering 185: 17–25. 10.1016/j.jfoodeng.2016.03.029.

[crf370315-bib-0098] Timms, R. E. 1984. “Phase Behaviour of Fats and Their Mixtures.” Progress in Lipid Research 23, no. 1: 1–38. 10.1016/0163-7827(84)90004-3. 6399934

[crf370315-bib-0099] Toro‐Vazquez, J. F. , M. Briceño‐Montelongo , E. Dibildox‐Alvarado , M. Charó‐Alonso , and J. Reyes‐Hernández . 2000. “Crystallization Kinetics of Palm Stearin in Blends With Sesame Seed Oil.” Journal of the American Oil Chemists' Society 77, no. 3: 297–310. 10.1007/s11746-000-0049-x.

[crf370315-bib-0100] Toro‐Vazquez, J. F. , and E. Dibildox‐Alvarado . 1997. “Parameters That Determine Tripalmitin Crystallization in Sesame Oil.” Journal of Food Lipids 4, no. 4: 269–282. 10.1111/j.1745-4522.1997.tb00099.x.

[crf370315-bib-0101] Ueno, S. , A. Minato , H. Seto , Y. Amemiya , and K. Sato . 1997. “Synchrotron Radiation X‐ray Diffraction Study of Liquid Crystal Formation and Polymorphic Crystallization of SOS (*sn*‐1,3‐Distearoyl‐2‐oleoyl Glycerol).” The Journal of Physical Chemistry B 101, no. 35: 6847–6854. 10.1021/jp9715639.

[crf370315-bib-0102] Valeri, D. , and A. J. A. Meirelles . 1997. “Viscosities of Fatty Acids, Triglycerides, and Their Binary Mixtures.” Journal of the American Oil Chemists' Society 74, no. 10: 1221–1226. 10.1007/s11746-997-0048-6.

[crf370315-bib-0103] van Herck, J. , M. V. Gil , K. M. Jablonka , et al. 2024. “Assessment of Fine‐Tuned Large Language Models for Real‐World Chemistry and Material Science Applications.” Chemical Science 16, no. 2: 670–684. 10.26434/chemrxiv-2024-mm31v. 39664810 PMC11629507

[crf370315-bib-0104] Van Langevelde, A. , K. Van Malssen , R. Driessen , et al. 2000. “Structure of C * _n_ * C * _n_ * _+2_ C * _n_ * ‐type (*n* = even) β′‐triacylglycerols.” Acta Crystallographica Section B Structural Science 56, no. 6: 1103–1111. 10.1107/S0108768100009927. 11099979

[crf370315-bib-0105] van Malssen, K. , R. Peschar , C. Brito , and H. Schenk . 1996. “Real‐time X‐ray Powder Diffraction Investigations on Cocoa Butter. III. Direct β‐crystallization of Cocoa Butter: Occurrence of a Memory Effect.” Journal of the American Oil Chemists' Society 73, no. 10: 1225–1230. 10.1007/BF02525450.

[crf370315-bib-0106] Van Mechelen, J. B. , R. Peschar , and H. Schenk . 2006. “Structures of Mono‐unsaturated Triacylglycerols. II. The β2 Polymorph.” Acta Crystallographica Section B: Structural Science 62, no. 6: 1131–1138. 10.1107/S0108768106037086. 17108668

[crf370315-bib-0107] Walstra, P. 2002. Physical Chemistry of Foods. CRC Press. 10.1201/9780203910436.

[crf370315-bib-0108] Wesdorp, L. H. 1990. Liquid–Multiple Solid Phase Equilibria in Fats: Theory and Experiments. CRC Press. 10.1201/b12883-11.

[crf370315-bib-0109] Wright, A. J. , S. S. Narine , and A. G. Marangoni . 2000. “Comparison of Experimental Techniques Used in Lipid Crystallization Studies.” Journal of the American Oil Chemists' Society 77, no. 12: 1239–1242. 10.1007/s11746-000-0194-2.

[crf370315-bib-0110] Zéberg‐Mikkelsen, C. K. , and E. H. Stenby . 1999. “Predicting the Melting Points and the Enthalpies of Fusion of Saturated Triglycerides by a Group Contribution Method.” Fluid Phase Equilibria 162, no. 1–2: 7–17. 10.1016/S0378-3812(99)00171-5.

[crf370315-bib-0111] Zwietering, M. H. , I. Jongenburger , F. M. Rombouts , and K. van't Riet . 1990. “Modeling of the Bacterial Growth Curve PUm.” Applied and Environmental Microbiology 56, no. 6: 00. https://journals.asm.org/journal/aem. 10.1128/aem.56.6.1875-1881.1990 PMC184525 16348228

